# Redox-Triggered Proton Transfer in Synthetic Models for Non-Heme Diiron Enzymes: Spectroscopic, Computational, and Mechanistic Insights

**DOI:** 10.1021/acs.inorgchem.6c04733

**Published:** 2026-09-25

**Authors:** Justin L. Lee, Saborni Biswas, Victoria F. Oswald, Joseph W. Ziller, A. S. Borovik, Emile L. Bominaar, Michael P. Hendrich

**Affiliations:** † Department of Chemistry, Department of Chemistry, 6612 Carnegie Mellon University, 4400 Fifth Avenue, Pittsburgh, Pennsylvania 15213, United States; ‡ Department of Chemistry, 8788 University of California, 1102 Natural Sciences II, Irvine, California 92697, United States

## Abstract

The transformations that occur at metalloenzyme active sites during catalysis are inextricably coupled to secondary-sphere interactions with surrounding amino acids and water molecules. Probing the elemental steps of these in vitro reactions remains a formidable challenge; their inherent catalytic velocity frequently prevents the isolation or detection of transient intermediates. Mapping distinct oxidation events, coupled with proton transfer steps, provides a clear pathway to elucidating these overarching catalytic mechanisms. High-valent iron-oxido and hydroxido species are key intermediates in oxidative processes catalyzed by nonheme iron proteins. Here, we employ structurally characterized bioinspired diiron complexes to systematically probe these elemental processes. We have spectroscopically characterized six distinct oxidation and protonation states of a single, asymmetric diiron core. Density functional theory calculations corroborate these findings, demonstrating strong agreement with the experimental electronic parameters and provide insight into the energetics governing individual electron and proton transfers. Reactivity profiling demonstrates that the synthesized Fe^III^Fe^IV^ complex efficiently abstracts a hydrogen atom from a phenolic substrate via a proton-coupled electron transfer (PCET) pathway that takes advantage of secondary sphere hydrogen bond interactions provided by the ligand framework. This process mimics the native tyrosyl radical generation observed in intermediate X of RNR, a key enzyme in DNA biosynthesis.

## Graphical abstract

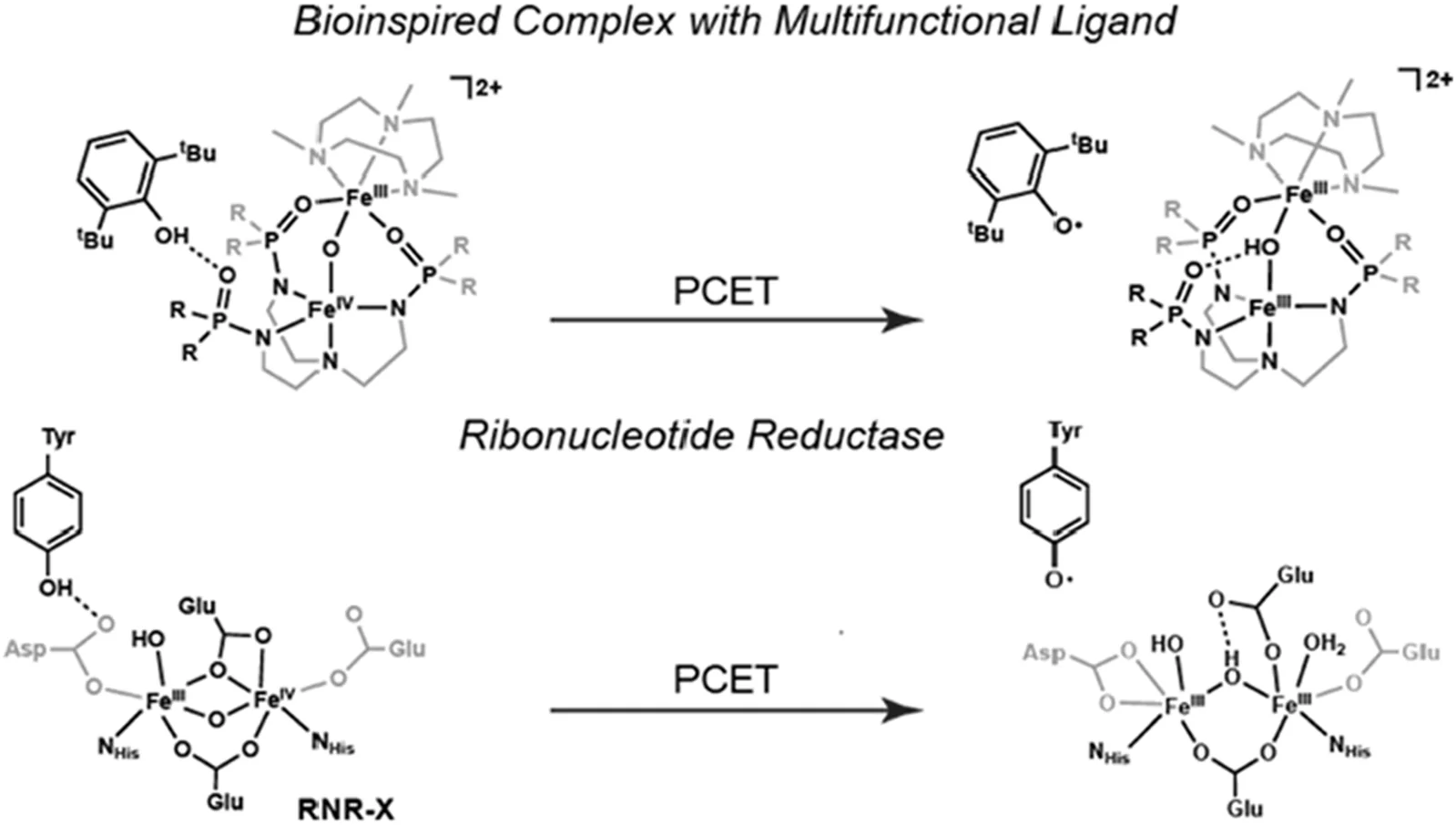

## Introduction

Diiron metalloproteins perform diverse biological functions, including O_2_ binding (hemerythrin), O_2_ activation (methane monooxygenase and the R2 subunit of ribonucleotide reductase [RNR R2]), NO/O_2_ reduction (flavin diiron proteins), and fatty acid oxidation (Δ^9^-desaturase). Oxidation of these di-Fe centers is often accompanied by structural changes, including proton transfer to or from coordinated ligands (e.g., OH^–^ and H_2_O), though such proton/electron transfer steps are difficult to evaluate experimentally. One example is RNR R2, in which the resting di-Fe^II^ state is converted to an Fe^III^Fe^IV^ intermediate (RNR-*X*), which cleaves the O–H bond of a distant tyrosine residue (Y122, ∼7 Å away) to generate a tyrosyl radical. The radical initiates a long-range electron transfer (>35 Å) to the R1 subunit, enabling the reduction of ribonucleotides to deoxyribonucleotides. A consensus structure for RNR-*X* ^1^ has two inorganic O-donors: a μ-O^2–^ between the irons and a terminal OH^–^ at the Fe^III^ site (Figure 1). The catalytic cycles of both heme (e.g., cytochrome P450) and nonheme (e.g., TauD) iron enzymes capable of H atom abstraction generally employ intermediates that have a terminal high-valent Fe-oxido moiety capable of direct interaction with the substrate. In contrast, the consensus structure of RNR-*X* has neither a terminal high-valent Fe-oxido moiety nor an oxido group that directly interacts with Y122, so it is unclear how the diiron cluster of RNR R2 can abstract an H atom from this residue within the secondary coordination sphere (Figure 1).

**Figure 1:**
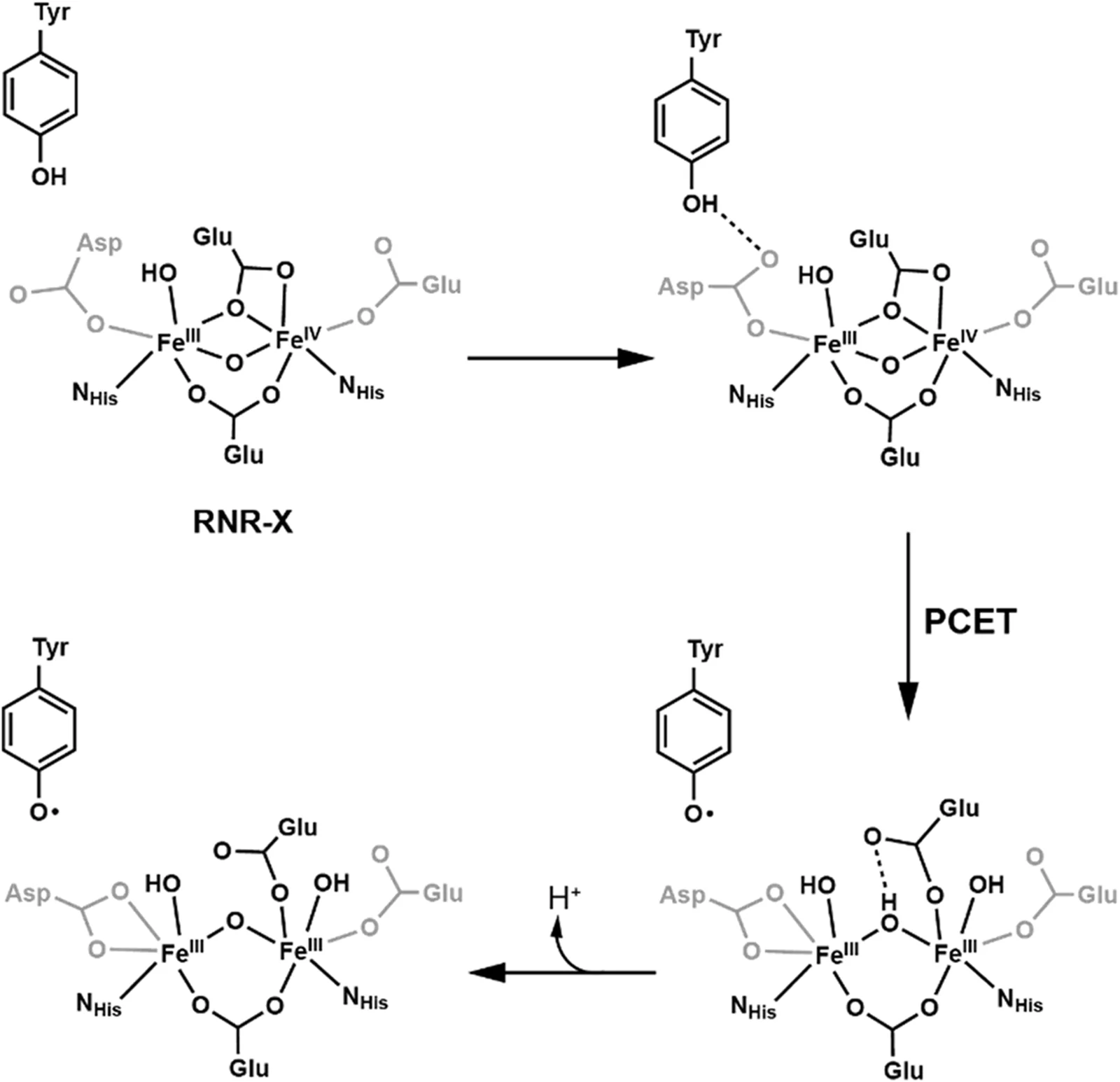
Our proposed chemical structure and reaction of Intermediate X in RNR R2.

To better understand the process of H atom abstraction by diiron enzymes, we previously reported a diiron complex with inequivalent coordination sites, in which one iron is bound to the tripodal ligand [poat]^3–^ (*N*,*N*′,*N*″-[nitrilotris­(ethane-2,1-diyl)]­tris­(*P*,*P*-diphenylphosphinic amido)) and the other to the macrocyclic ligand [TMTACN]^0^ (1,4,7-trimethyl-1,4,7-triazacyclononane).^2^ Remarkably, two of the phosphinic amido oxygen atoms from the [poat]^3–^ ligand also bind to the [FeTMTACN] unit, and complete its coordination sphere with a (hydr)­oxido bridging ligand. The third phosphinic amido oxygen atom can form an intramolecular hydrogen bond (H-bond) with the μ–OH^–^ bridge. The precursor for the diiron complexes studied here is the mixed-valence triply bridged [(TMTACN)­Fe^II^–(μ–OH)–Fe^III^poat]^+^ complex (**1**, Figure 2) that resembles the diiron cores found in biology. Through reductions, oxidations, and (de)­protonations of precursor **1**, the spectroscopic characterization of the six dinuclear complexes of Figure 2 will be presented. In addition, the characterization of two Fe-poat monomeric complexes (**2**, **3**, Figure 2), the Fe^II^ complex being new, will be reported that are relevant to the formation of the diiron complexes.

**Figure 2:**
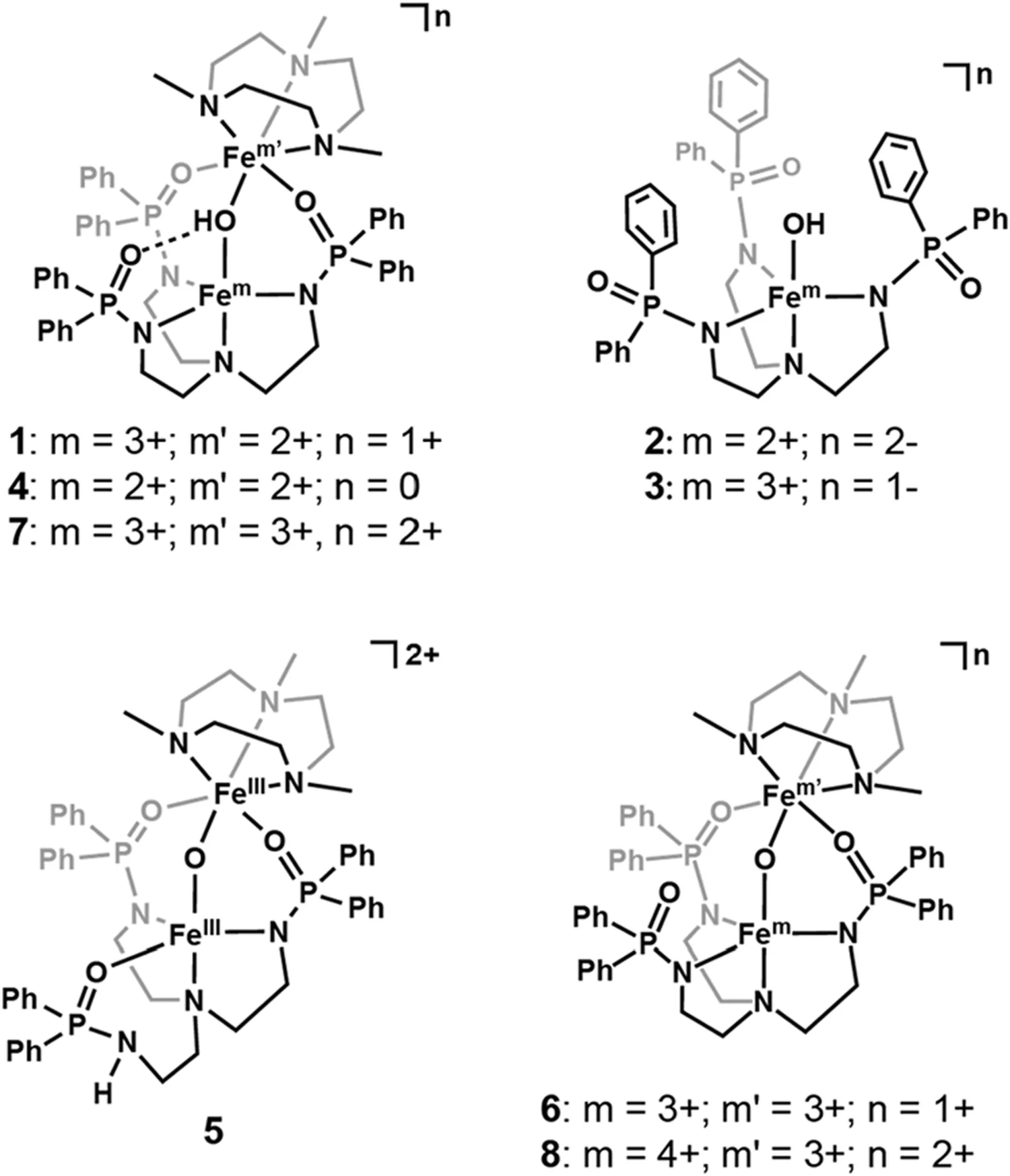
Structures of the mono- and di-iron complexes used in this study.

Biomimetic diiron complexes usually employ symmetric ligands presenting equivalent iron coordination spheres. Herein, the use of TMTACN and [poat]^3–^ ligands give different iron coordination environments with 6- and 5-coordinate geometries, respectively. Formation of the complexes are corroborated with spectroscopic analyses that were critical for identification of the species, their electronic properties, and assessment of purity. Our previous study showed that the inequivalence of iron sites in these complexes was readily apparent in the Mössbauer spectra and that this bimetallic complex can access the range of oxidation states observed for the diiron cluster in RNR. Furthermore, reaction of a tyrosine-like substrate with the Fe^III^Fe^IV^ complex generated oxidized products consistent with the formation of a phenoxyl radical reminiscent of the tyrosine radical in RNR. In-depth interpretation of Mössbauer results (including variable magnetic field analysis) and further connections to DFT calculations were beyond the scope of our initial report.^2^ Herein, we present the data that were used to establish the Fe oxidation states, the di-Fe site differentiation, and the protonation state of the bridging O­(H) unit. Starting with the precursor complex **1**, we followed the stepwise oxidation/reduction and accompanying changes in protonation of the [Fe–(O­(H))–Fe] core with spectroscopy and DFT calculations. These efforts resulted in a detailed characterization of a series of oxidation and protonation states, comprised of six diiron complexes supported by the same asymmetric ligand framework. The Mössbauer and EPR data also allowed us to determine the diiron exchange-coupling constant of the complexes from which the protonation state of the bridging ligand could be inferred, as well as pinpointing which Fe center undergoes oxidation or reduction. In addition, we provide Mössbauer parameters for two relevant monomeric precursors (**2**, **3**) and show the large differences in their properties compared to the [Fe^m+^poat] unit found in the di-Fe systems. Furthermore, from DFT calculations we were able to probe the reaction mechanism of the Fe^III^–O–Fe^IV^ complex with phenols, one that is directly related to the one found in RNR. Our mechanism suggests that the oxido bridging ligand in the Fe^III^–O–Fe^IV^ complex is competent to act as an entry point for receiving the proton during H atom abstraction, even though the di-Fe core cannot directly access the phenolic substrate, and thus constitutes an alternative possible reaction pathway for RNR-*X*.

## Results

### Molecular Structures for K_2_[**2**] and K­[**3**]

Addition of one equivalent of water and one equivalent of potassium hydride to K­[Fe^II^poat][Bibr ref3] allowed for the isolation of orange crystals of K_2_[**2**]. The molecular structure was determined by X-ray diffraction, which revealed the salt crystallized as a cluster with two anions of [Fe^II^poat­(OH)]^2–^ bound to four potassium ions, K_4_[**2**]_2_·4THF (Figure S1). Each Fe adopts a trigonal bipyramidal geometry (τ_5_ = 0.689), with a hydroxido ligand skewed for its axial position because of coordination to three potassium cations. The Fe–O bond distance of 2.032 Å is consistent with other reported mononuclear Fe­(II)-hydroxido complexes.^4,5^ The preparation and molecular structure of K­[Fe^III^poat­(OH)] have previously been reported.^6^ Briefly, this salt also crystallized as a cluster with two complex anions and only two potassium ions (i.e., {K­[**3**]}_2_), with the Fe–O­(H) bond distance shortened to 1.896 Å upon oxidation. In the solid-state structures of both salts, the binding of the P=O ligands to the potassium counterions disrupts their intramolecular H-bonding interactions with the hydroxido ligand.

### Mössbauer Properties for **2**

Low temperature (4.2 K) Mössbauer spectra at variable magnetic fields of K_2_[**2**] with 18-crown-6 (18-c-6) in 1:1 DMF:THF are shown in Figure 3. In a magnetic field of 0.05 T (Figure 3A), the complex showed a single quadrupole doublet with parameters δ = 1.03 mm/s and Δ*E*_Q_ = −3.05 mm/s (Table 1). These parameters are typical of high-spin Fe^II^ complexes.^7^ For all complexes in Table 1, either a + or – sign for Δ*E*_Q_ will be shown if it could be determined from the spectra recorded in a magnetic field, otherwise no sign is given. At higher magnetic fields, the complex displayed two overlapping magnetic hyperfine patterns from two high-spin Fe^II^ species in a ratio of 70:30. The simulations indicate that both species have approximately the same δ and Δ*E*_Q_ values but different **A** and *D* values. The parameters of the majority species are listed in Table 1. Two independent preparations showed the same two species in the same ratio. The similarity of the δ and Δ*E*_Q_ values indicates that the iron primary coordination spheres of the two species are similar. Consistent with the large positive *D* values of both species, the sample did not exhibit EPR signals in parallel mode. The low temperature Mössbauer spectra of a powder sample of K_2_[**2**] revealed a species different from either of the solution species. The 7 T spectrum is shown in Figure 3E for comparison and the full set of simulations are shown in Figure S13. The isomer shift is the same as in solution, however all other parameters have changed significantly (Table 1), indicating that the solution- and solid-state structures are different.

**Figure 3:**
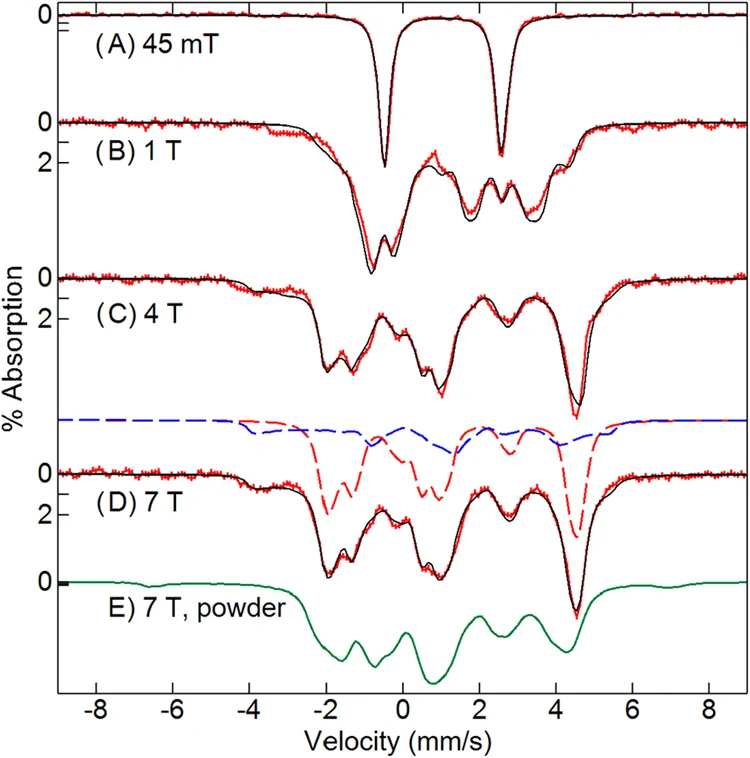
Mössbauer spectra (red traces, A–D) of **2** in 1:1 DMF:THF recorded at 4.2 K and the magnetic fields as listed. The black traces are global least-squares fits for two S = 2 Fe^2+^ species. The parameters for the major species (70%) are given in Table 1. The minor species (30%) has the parameters: *S* = 2, *D* = +3.7 cm^–1^, *E*/*D* = 0.2, A = (−18, −8, −4) *T*, δ = 1.08 mm/s, Δ*E*_Q_ = −3.13 mm/s. For the 7 T spectrum (D), the component spectra giving the sum are shown (major, red dash; minor, blue dash). The neat powder spectrum of the complex (E) was recorded under the same conditions as *D*.

**Table 1:** Experimental and DFT Calculated (in Parentheses) Parameters for the Complexes of This Study

complex	*S*	*D* (cm^–1^)	*E/D*	*J* (cm^–1^)	δ (mm/s)	Δ*E*_*Q*_ ^b^ (mm/s)	η	**A** ^c^ (*T*)
**2** (OH) Fe^II^	2	8	0.03		1.03(1.04)	–3.05(−3.57)	0.4(0.4)	–12, −12, −22
**2** (OH) powder^a^	2	–18	0.30		1.03	+2.92(+3.32)	0.7(0.4)	–21, −38, −10
**3** (OH) Fe^III^	5/2	–1.2	0.23		0.29(0.33)	+1.06(+0.64)	0.1(0.4)	–20
**4** (OH)	0			20				
Fe^II^poat	2	3.7	0.14	(26)	1.03(1.03)	+3.38(+3.65)	0.7(0.4)	–40, −21, −3
Fe^II^TMTACN	2	5.6	0.20		1.22(1.21)	+2.38(+2.78)	0.5(0.1)	–38, −13, −2
**1** (OH)	1/2			40				
Fe^III^poat	5/2	3.1	0.20	(48)	0.36(0.34)	–0.58(−0.68)	0(0.2)	–20
Fe^II^TMTACN	2	21	0.08		1.19(1.19)	+2.77(+4.20)	1(0.6)	–9, −7, −9
**7** (OH)	0			50				
Fe^III^poat	5/2			(70)	0.36(0.33)	–0.61(−0.73)	0.2(0.8)	–20
Fe^III^TMTACN	5/2				0.47(0.46)	–1.10(−0.90)	0.4(0.1)	–21
**5** (O)	0			>100				
Fe^III^Hpoat	5/2			(190)	0.45(0.38)	1.15(−0.73)	0.9(0.7)	–20
Fe^III^TMTACN	5/2				0.51(0.47)	–1.75(−1.83)	0.2(0.1)	–21
**6** (O)	0			>100				
Fe^III^poat	5/2			(220)	0.38(0.33)	1.16(+1.00)	0.8(0.31)	–20
Fe^III^TMTACN	5/2				0.53(0.49)	–1.86(−2.07)	0.1(0.09)	–21
**8** (O)	1/2			>100				
Fe^IV^poat	2			(150)	0.00(0.00)	+0.58(+1.04)	0.8(0.2)	–13, −17, −23
Fe^III^TMTACN	5/2				0.47(0.47)	–1.11(−1.00)	0.6(0.3)	–21

a Δ*E*_Q_ and η from DFT results for XRD structure.

b A sign is stated for the experimental value of Δ*E*_Q_ if it could be determined. The DFT value uses a conversion factor of −1.65 mm·s^–1^/AU.

c Single values imply an isotropic A tensor.

A gas-phase DFT optimization of **2** computed the structure shown in Figure 4A. Steric repulsions between the phenyl groups of [poat]^3–^ resulted in a preferred conformation in which one aromatic ring of each ligand arm is positioned parallel to the Fe^II^–O­(H) bond and the intersection of the phenyl planes of each arm coincides with the P=O bond. The optimized structure also shows that the three O=P bonds roughly align with the plane formed by the equatorial N-atoms of the phosphinic amido groups. We found other stable conformations in our calculations, albeit at higher energies (Figure S2). For all these optimized structures, none of the O atoms in [poat]^3–^ engage in an intramolecular H-bond with the hydroxido ligand, as in the diiron complex discussed below, even when the geometry optimization was initiated using a structure in which the H-bond was present. We found that the calculated Mössbauer parameters δ and Δ*E*_Q_ for the optimized structure of Figure 4A agreed with the experimental values of the solution sample (Table 1).

**Figure 4:**
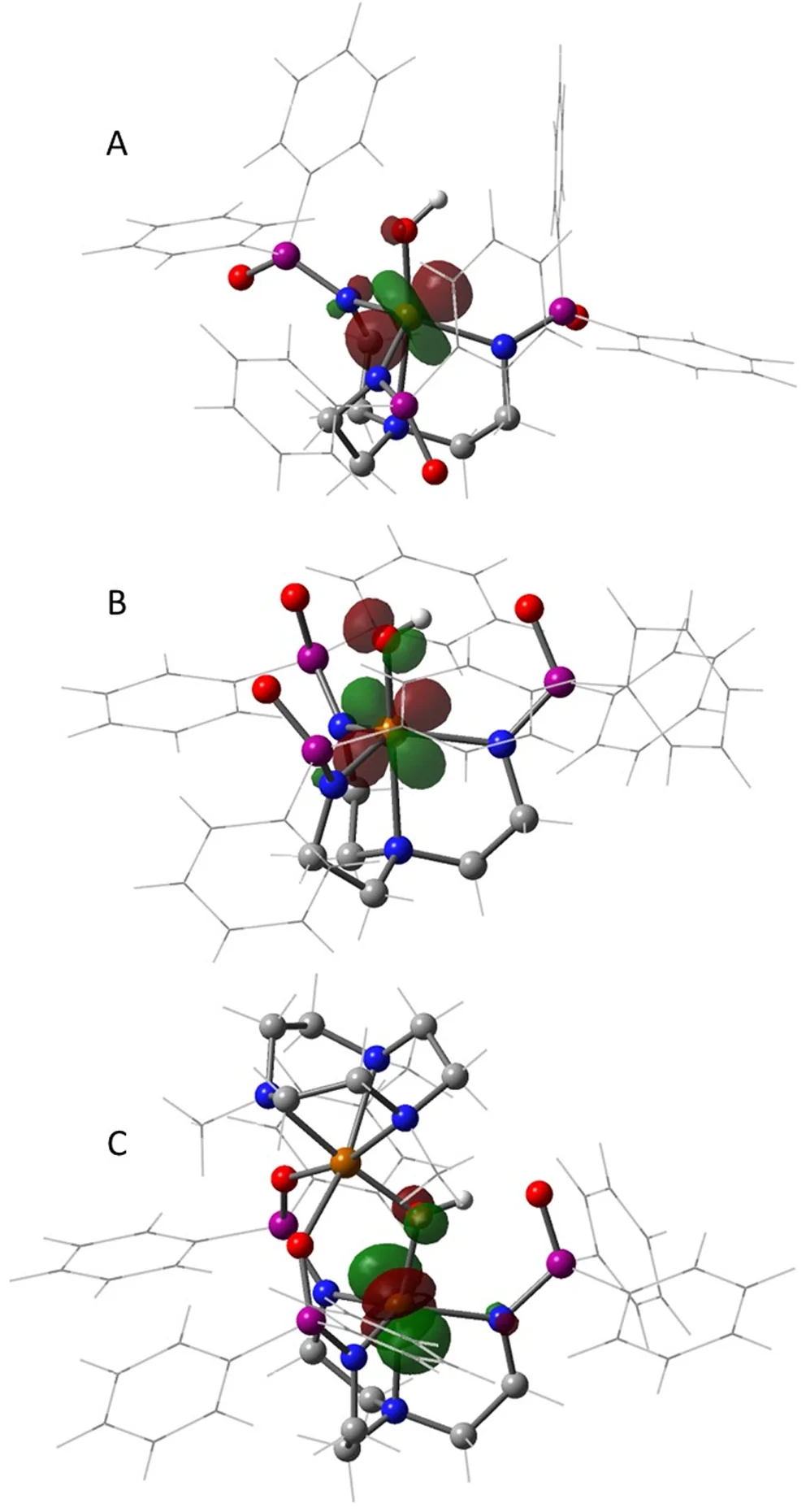
(A) DFT optimized structure of **2**, (B) XRD determined structure of [Fe^II^poat­(OH)]^2–^ fragment within the solid K_2_[Fe^II^poat­(OH)]_2_ cluster, and (C) the DFT optimized structure of the diferrous complex **4**. Superimposed are the contour plots of the doubly occupied 3*d* iron orbital of the [Fe^II^poat]^−^ moiety. For clarity, only the H atoms on the hydroxido ligands are shown. Color code: C (gray), N (blue), O (red), P (violet), Fe (brown).

### Mössbauer and EPR Properties of **3**

Low temperature (4.2 K) Mössbauer spectra of **3** with 18-c-6 in 1:1 DMF:THF (Figure 5A,B) were collected at variable magnetic field and the least-squares simulations shown produced the parameters listed in Table 1. An EPR spectrum of the same complex measured at 12 K (Figure 5C) revealed two species with the majority species (85%) having electronic parameters which agreed with the Mössbauer results. The minority species (15%) is distinguished by its smaller *E*/*D* value of 0.13 which is not resolved in the Mössbauer spectra; this species will not be discussed further. A spectrum of a powder sample of K­[Fe^III^poat­(OH)] gave broad features which were difficult to interpret. A gas-phase DFT optimization of the [Fe^III^poat­(OH)]^−^ complex also gave a structure in which the conformations of the P=O groups are not proximal to the Fe^III^–OH unit, resulting in the lack of intramolecular H-bonds. The calculated Mössbauer parameters for the optimized structure agree with experimental values in solution (Table 1).

**Figure 5:**
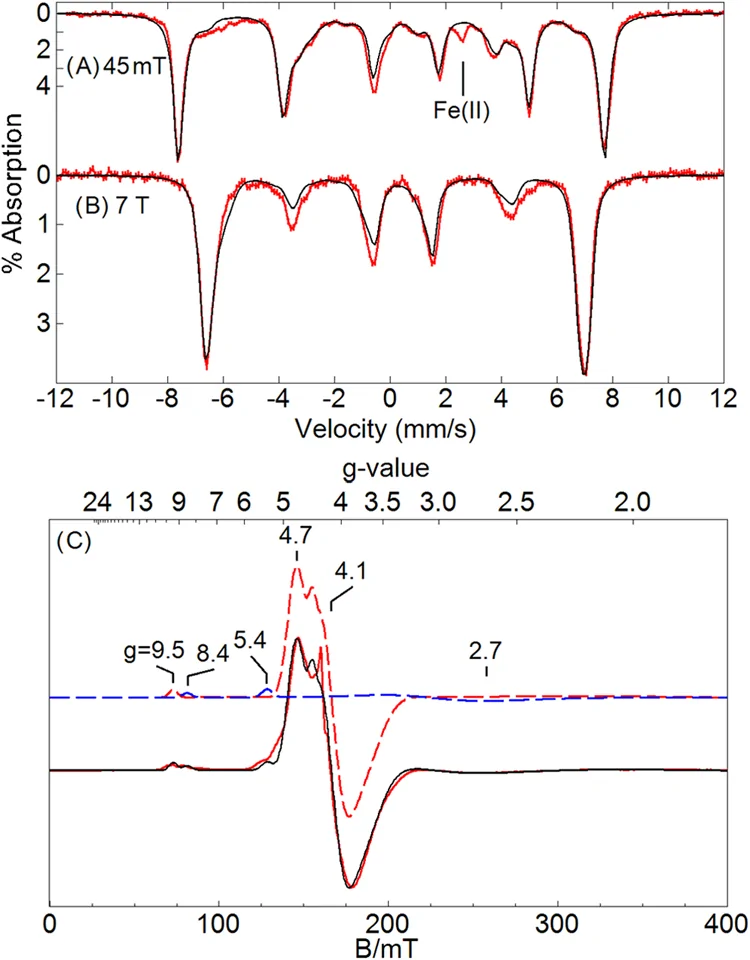
Mössbauer spectra (A and B, red traces) of **3** with 18-crown-6 in 1:1 DMF:THF recorded at 4.2 K and the magnetic fields as listed. The black traces are global least-squares fits for an *S* = 5/2 Fe^III^ species with the parameters given in Table 1. A minor amount of the precursor Fe^II^ complex gives a doublet with the line marked at 2.6 mm/s. (C, red trace) EPR spectrum (9.643 GHz) of **3** at 12 K. The black trace is a simulation with two species. The parameters of the major species (85%, red dash) are listed in Table 1; the minor species (15%, blue dash) has *E*/*D* = 0.13 and is not resolved in the Mössbauer spectra.

### Mössbauer and EPR Properties of [(TMTACN)­Fe^II^−(μ−OH)−Fe^III^poat]^+^ (**1**)

Treating [Fe^II^TMTACN­(OTf)_2_] with **3** produced complex **1** which served as the synthon for all the diiron complexes of this study. An EPR spectrum of the complex^2^ displayed an *S* = 1/2 rhombic signal with *g* = (1.95, 1.50, 1.20) arising from antiferromagnetic coupling between a rhombic Fe^II^ (*S* = 2) center with *g*-values (2.04, 2.38, 2.53) and an Fe^III^ (*S* = 5/2) center with assumed *g*-values of ∼2.0. A determination of the magnitude of the exchange interaction from EPR data was not possible, owing to signal broadening at higher temperatures. Spin quantification of the signal gave an amount consistent with the concentration of the complex in solution.

Mössbauer spectra of **1** were recorded for variable magnetic fields and temperatures (Figure 6). The spectrum in zero magnetic field at 100 K (Figure 6A) showed two distinct quadrupole doublets with parameters typical of Fe^III^ and Fe^II^ sites (Table 1). The global fit to the variable magnetic field and temperature spectra gave an exchange-coupling constant of *J* = 40 cm^–1^; the magnitude of this exchange coupling is indicative of a hydroxido bridge between the iron sites.^8^ The Mössbauer results agree with the EPR data, showing the formation of an *S* = 1/2 system via an antiferromagnetic coupling between high-spin Fe^III^ and Fe^II^ sites.

**Figure 6:**
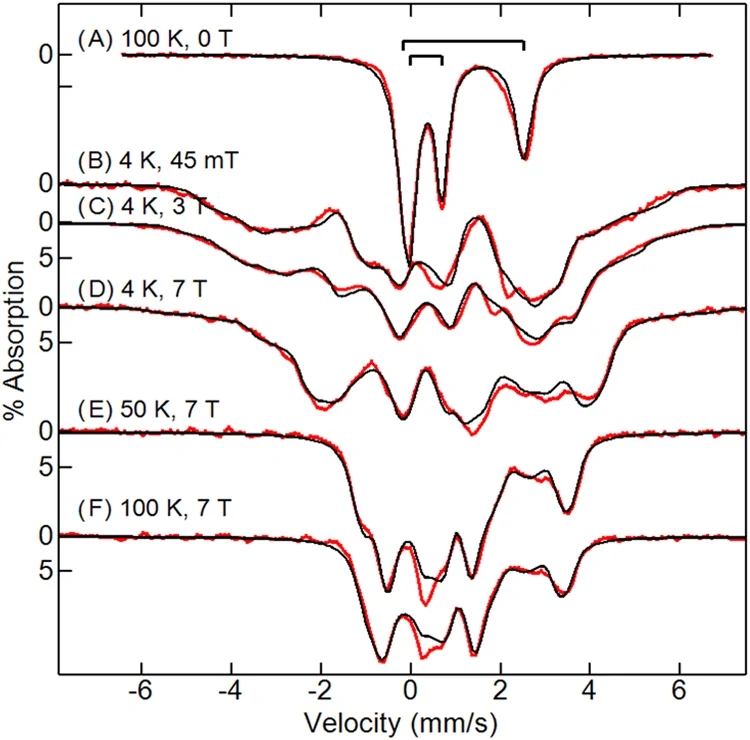
Mössbauer spectra (red traces) of **1** in butyronitrile (PrCN) for the magnetic fields and temperatures as listed. The black traces are global least-squares fits for antiferromagnetically coupled Fe^II^ *S* = 2 and Fe^III^ *S* = 5/2 sites with *J* = 40 cm^–1^ using the parameters in Table 1.

The bond lengths of **1** determined by XRD methods further support that the Fe center coordinated to the anionic N-donors of [poat]^3–^ is ferric (denoted [Fe^III^poat]) and the other Fe center is ferrous (denoted [Fe^II^TMTACN]). The isomer shift of 0.36 mm/s for the 5-coordinate [Fe^III^poat] site is lower than the range of nonheme 6-coordinate Fe^III^ species (0.4–0.6 mm/s)^7^ but close to that measured for the mononuclear complex **3**. The smaller coordination number lowers the electron density in the 3*d* orbitals, causing less shielding of the nuclear charge, contraction of *s*-orbitals, and an increase in electron density at the nucleus, thereby lowering the isomer shift relative to six-coordinate complexes. The unconstrained DFT optimization of **1** gave a molecular structure in close agreement with the structure obtained by XRD methods. The calculated Mössbauer and exchange parameters of the optimized structure mostly agreed with experiment, supporting our assignment of parameters to the [Fe^III^poat] and [Fe^II^TMTACN] sites (Table 1). DFT calculations found that the exchange parameter was influenced by the hydrogen bond of the bridging hydroxido ligand to the O=P group in one of the [poat]^3–^ arms (Figure S3). The value for Δ*E*_Q_ for the [Fe^II^TMTACN] site from DFT (+4.20 mm/s) has the same sign as observed experimentally (+2.77 mm/s) but is significantly higher in magnitude, unlike in its diferrous analog (see below) where experiment and theory approximately agree. Following the work of Goldberg,^9^ we pursued the possibility that the lower experimental value of Δ*E*_Q_ may result from a 5-coordinate Fe^II^ site in solution. However, this premise was ruled out because Goldberg’s 5-coordinate species have δ values <1 mm/s that are well below those of both **1** and the 6-coordinate diferrous species **4**. The increased oxidation of the iron across the bridge, which has minor influence on the experimental value for Δ*E*_Q_ for the TMTACN site, has apparently a large but artificial influence on the value of Δ*E*_Q_ obtained from DFT.

### Mössbauer and EPR Properties of [(TMTACN)­Fe^II^– (μ–OH)–Fe^II^poat] (**4**)

Treating **1** with 1 equiv of CoCp*_2_ afforded complex **4**. The Mössbauer spectrum of **4** measured at 4.2 K and 45 mT showed a diamagnetic pattern with two distinct quadrupole doublets with parameters indicative of a diferrous species (Figure 7A, and Table 1). Based on the comparison with the mononuclear complex **2**, the doublet with δ = 1.03 mm/s, Δ*E*_Q_ = +3.38 mm/s was assigned to the [Fe^II^poat] site, and the doublet with δ = 1.22 mm/s, Δ*E*_Q_ = +2.38 mm/s was assigned to the [Fe^II^TMTACN] site. Moreover, the Mössbauer parameters of the [Fe^II^TMTACN] site are close to those found for the ferrous site of the precursor complex **1**. Based on its lower coordination number, the isomer shift of the [Fe^II^poat­(OH)] site (1.03 mm/s) is expected to be lower than that of [Fe^II^TMTACN] site (1.22 mm/s). Six-coordinate nonheme Fe^II^ centers have a range of isomer shifts of 1.1–1.3 mm/s^7^ and their quadrupole splitting values can vary appreciably with sample temperature. Thus, the quadrupole splitting for both Fe centers were determined in the absence of a magnetic field over the range of 4 to 130 K (Table S6), and these values were used for simulations of the temperature dependent spectra (Figure 7B–D) in high magnetic field to determine the exchange coupling between the Fe^II^ sites. The simulations of the experimental spectra at a magnetic field of 7 T indicate an antiferromagnetic coupling of *J* = 20 cm^–1^ between the two high-spin Fe^II^ centers. The magnitude of the exchange coupling is consistent with a hydroxido bridge between the Fe^II^ centers and thus supports our formulation of complex **4**.

**Figure 7:**
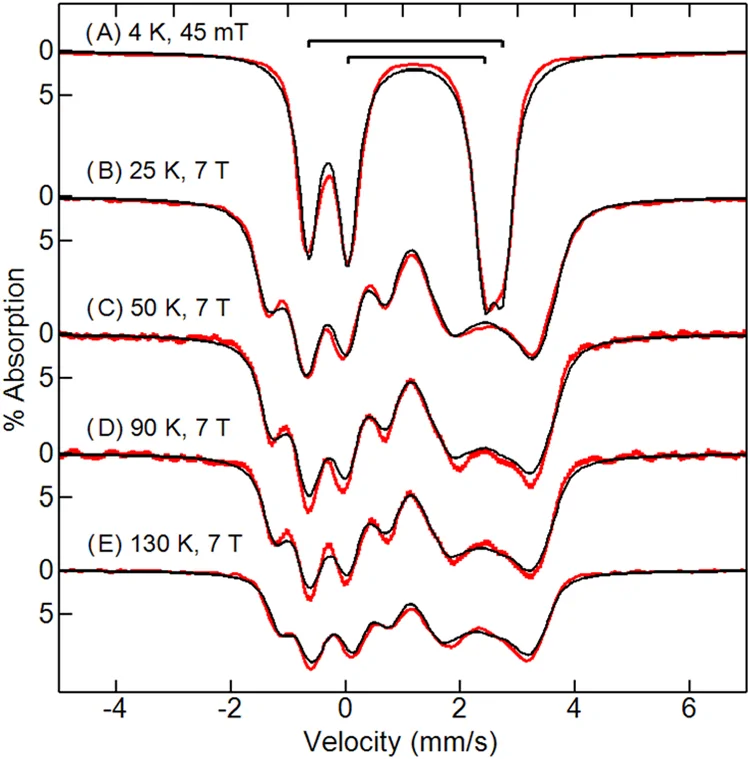
Mössbauer spectra (red traces) of **4** in PrCN for the temperatures and magnetic fields as listed. The black traces are global least-squares fits for two antiferromagnetically coupled *S* = 2 Fe^2+^ sites (*J* = 20 cm^–1^) using the parameters in Table 1.

The calculated Mössbauer parameters of the optimized DFT structure of **4** are in good agreement with those from experiment, suggesting that the structure of the reduced complex closely resembles the optimized DFT structure (Figure 4C). The positive Δ*E*_Q_ value observed and calculated for the [Fe^II^poat­(OH)] site in **4** arises from the oblate (“xy like”) shape of the doubly occupied 3*d* orbital (Figure 4). Additional details from DFT calculations are given near Figure S4. Several attempts to obtain single crystals of **4** suitable for XRD analysis were unsuccessful, preventing a detailed structural analysis for this complex.

### Mössbauer Properties of [(TMTACN)­Fe^III^–(μ-O)–Fe^III^Hpoat]^2+^ (**5**)

One-electron oxidation of **1** with [FeCp_2_]­OTf afforded the isolation of complex **5**. Our XRD studies found that [poat]^3–^ was protonated to [Hpoat]^2–^ upon oxidation, presumably with the concomitant formation of an Fe^III^–(O)–Fe^III^ core through an intramolecular proton transfer (PT) process (see Discussion section).^2^ The complex was studied with Mössbauer spectroscopy in both the solution and solid states. The solution and solid samples displayed nearly identical spectra indicating that the oxidized complex remains intact in solution. The solution spectrum recorded at 4.2 K in zero magnetic field (Figure 8A) shows two quadrupole doublets with parameters typical of high-spin ferric sites (Table 1). The isomer shift of the [Fe^III^TMTACN] site does not vary significantly from the other diferric complexes in this study, hence the outer doublet of δ = 0.51 mm/s, Δ*E*_Q_ = −1.75 mm/s is assigned to this site. Consequently, the inner doublet with δ = 0.45 mm/s, Δ*E*_Q_ = 1.15 mm/s is assigned to the [Fe^III^Hpoat] site. This isomer shift (0.45 mm/s) is significantly higher than those for the [Fe^III^poat] fragment in all other Fe^III^ complexes with this tripodal ligand (ranging from 0.29 to 0.35 mm/s). The structural studies revealed that this Fe^III^ site has an N_3_O_2_ primary coordination sphere, comprising of the [Hpoat]^2–^ ligand and a bridging oxido ligand (**5**, Figure 2). The coordination of the second O atom arises from the N-protonated phosphinic amide arm that binds to the Fe^III^ center through its P=O group. This binding mode results in a 7-member chelate ring and a large distortion in the trigonal plane which, in addition to the N_3_O_2_ sphere, could cause the higher isomer shift. The DFT calculated isomer shift from the optimized structure is consistent with a high value (0.38 mm/s), although not as high as experiment. Variable temperature spectra recorded in a 7 T magnetic field (Figure 8B–D) and their simulations gave an antiferromagnetic exchange-coupling constant of *J* > 100 cm^–1^. Consistent with the XRD results, the exchange-coupling constant indicated that the bridging hydroxido ligand has been converted to a bridging oxido. The optimized DFT structure agreed with this structural change and gave a DFT calculated value of *J* = 190 cm^–1^. All other DFT computed parameters are consistent with experimental values.

**Figure 8:**
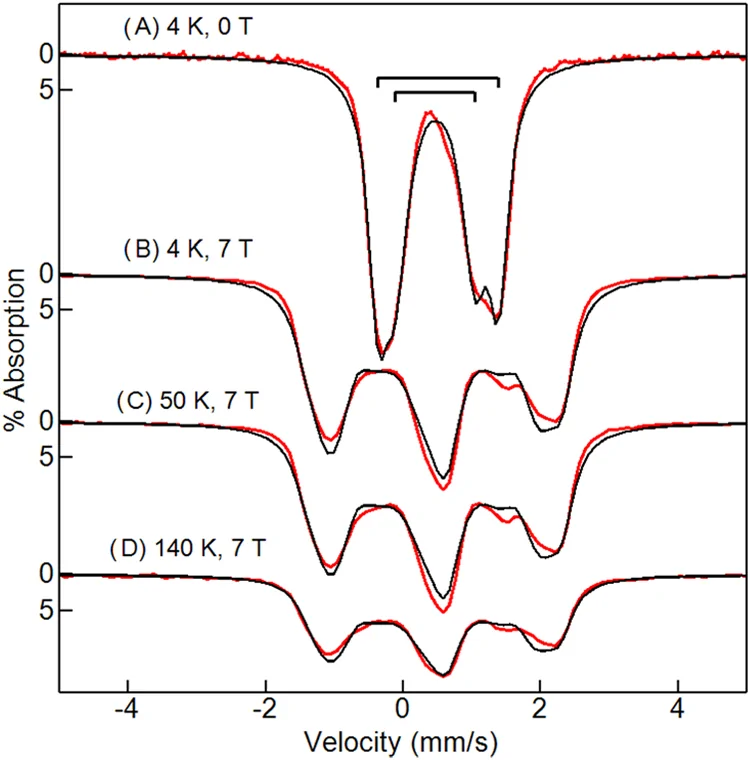
Mössbauer spectra (red traces) of **5** in PrCN recorded at the temperatures and magnetic fields as listed. The black traces are global least-squares fits for antiferromagnetically coupled Fe^III^ *S* = 5/2 sites with *J* > 100 cm^–1^ using the parameters in Table 1.

### Mössbauer Properties of [(TMTACN)­Fe^III^–(μ-O)–Fe^III^poat]^+^ (**6**)

The treatment of **5** with one equiv of triethylamine formed complex **6** whose molecular structure from XRD studies showed conversion to the fully deprotonated [poat]^3–^ ligand. Complex **6** was EPR silent and its Mössbauer spectrum recorded in zero-field at 4.2 K showed two quadrupole doublets typical of ferric sites (Figure 9A, and Table 1). The Mössbauer parameters of the [Fe^III^TMTACN] site are close to those of complex **5**, and consequently, the quadrupole doublet with δ = 0.38 mm/s, Δ*E*_Q_ = 1.16 mm/s is assigned to the [Fe^III^poat] site. Upon deprotonation, the isomer shift of this [Fe^III^poat] site returns to a value close to that found for complexes **1** and **3**. The Mössbauer parameters and the magnetic data (Figure 9) indicate that the Fe^III^ sites of this complex are antiferromagnetically coupled with *J* > 100 cm^–1^; DFT calculations gave an exchange-coupling constant of 220 cm^–1^. The magnitude of *J* is consistent with the presence of a bridging oxido ligand. The XRD structure, the optimized DFT structure, the experimental and DFT calculated Mössbauer parameters are all in good agreement.

**Figure 9:**
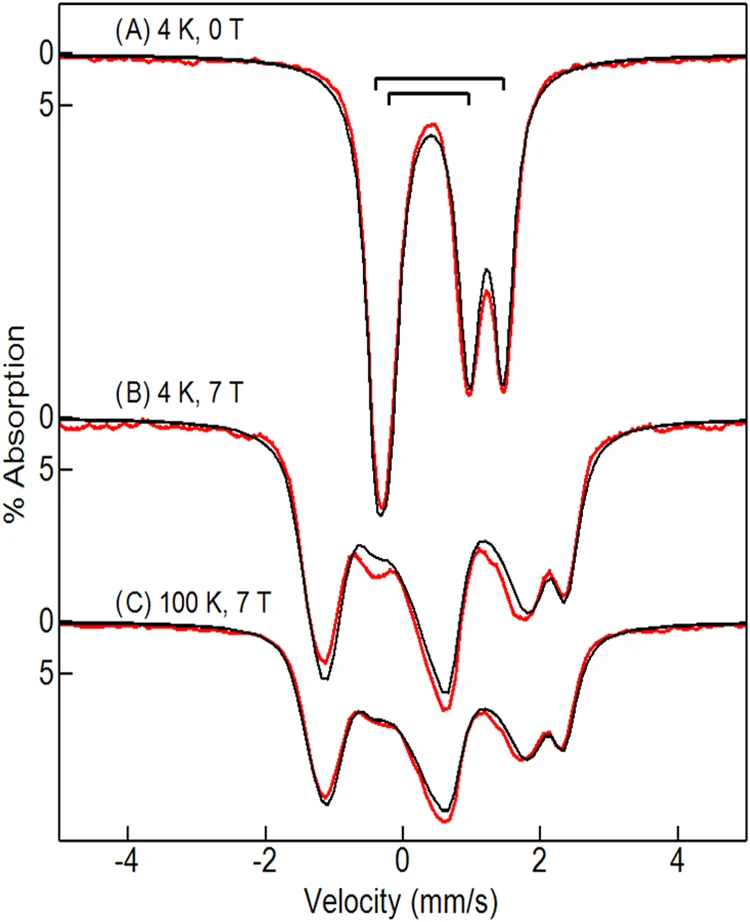
Mössbauer spectra (red traces) of **6** in PrCN recorded at the magnetic fields and temperatures as listed. The black traces are global least-squares fits for antiferromagnetic coupled Fe^III^ *S* = 5/2 sites with *J >* 100 cm^–1^ using the parameters in Table 1.

### Mössbauer and EPR Properties of [(TMTACN)­Fe^III^–(μ–OH)–Fe^III^poat]^2+^ (**7**)

The intriguing result that the one-electron oxidation of complex **1** triggers the intramolecular PT to form complex **5** motivated us to perform low-temperature studies of this reaction with the aim of possibly observing the thermally unstable intermediate with an Fe^III^–(OH)–Fe^III^ core. In fact, treating **1** in PrCN at −80 °C with 1 equiv of [FeCp_2_]­OTf produced a species that is formulated as complex **7**, which can also be generated from the reaction of [(TMTACN)­Fe^III^–(μ-O)–Fe^IV^poat]^2+^ (**8**) with phenolic substrates (*vide infra*). The Mössbauer spectrum of **7** shows two quadrupole doublets in low magnetic field at 4.2 K (Figure 10A) with parameters typical of high-spin ferric sites. Based on the other complexes in this study, the inner doublet of δ = 0.36 mm/s, Δ*E*_Q_ = −0.61 mm/s is assigned to the [Fe^III^poat] site, and the outer doublet of δ = 0.47 mm/s, Δ*E*_Q_ = −1.10 mm/s is assigned to the [Fe^III^TMTACN] site. Variable temperature spectra and simulations in a 7 T magnetic field (Figure 10B–E), and a global least-squares fit to the data gave an antiferromagnetic exchange-coupling constant of *J* = 50 cm^–1^.

**Figure 10:**
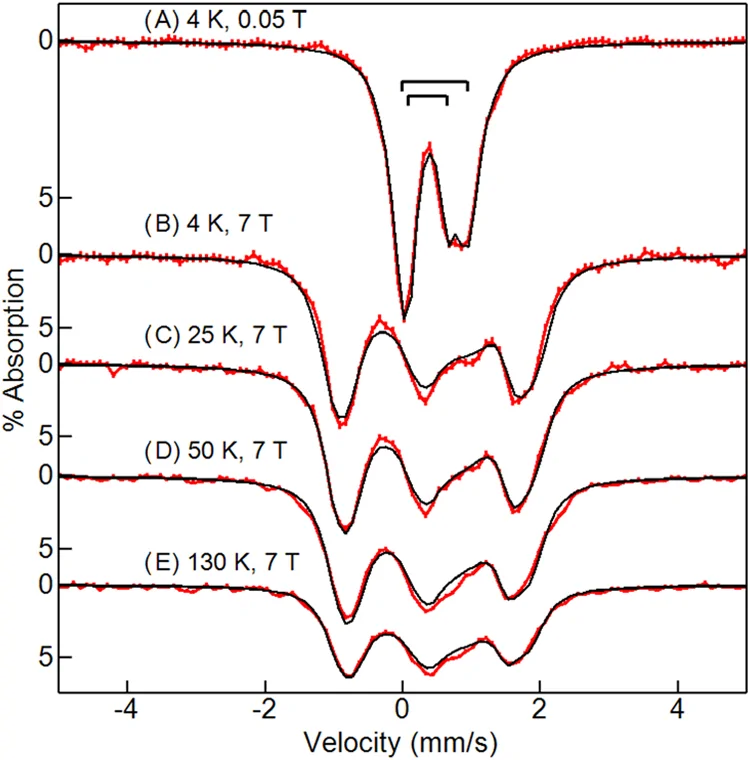
Mössbauer spectra (red traces) of **7** in PrCN at the temperatures and magnetic fields as listed. The black traces are global least-squares fits for antiferromagnetically coupled Fe^III^ *S* = 5/2 sites with *J* = 50 cm^–1^ using the parameters in Table 1.

Owing to the relatively small values of *D* for nonheme high-spin Fe^III^ sites, the integer-spin states of exchange coupled ferric sites generally have a small energy splittings (<0.3 cm^–1^) within the M_s_ substates of multiplets with *S* > 1. As a result, the detection of EPR signals is possible with a microwave field oscillating parallel to the static magnetic field (parallel-mode).^8,10^ For complex **7**, the magnitude of the *J*-value is sufficiently low that the *S* > 1 spin states are populated at temperatures less than 150 K. Therefore, parallel-mode EPR spectra of **7** were collected over a range of temperatures (Figure 11) and a signal near *g* = 12 was observed. The spectra are plotted for the same instrumental intensity scale. Simulations of this signal are shown for *J* = 45 cm^–1^ which quantitatively agree with the intensity of the species for the various temperatures. The signal is dominated by transitions from within the excited *S* = 2 coupled spin state.

**Figure 11:**
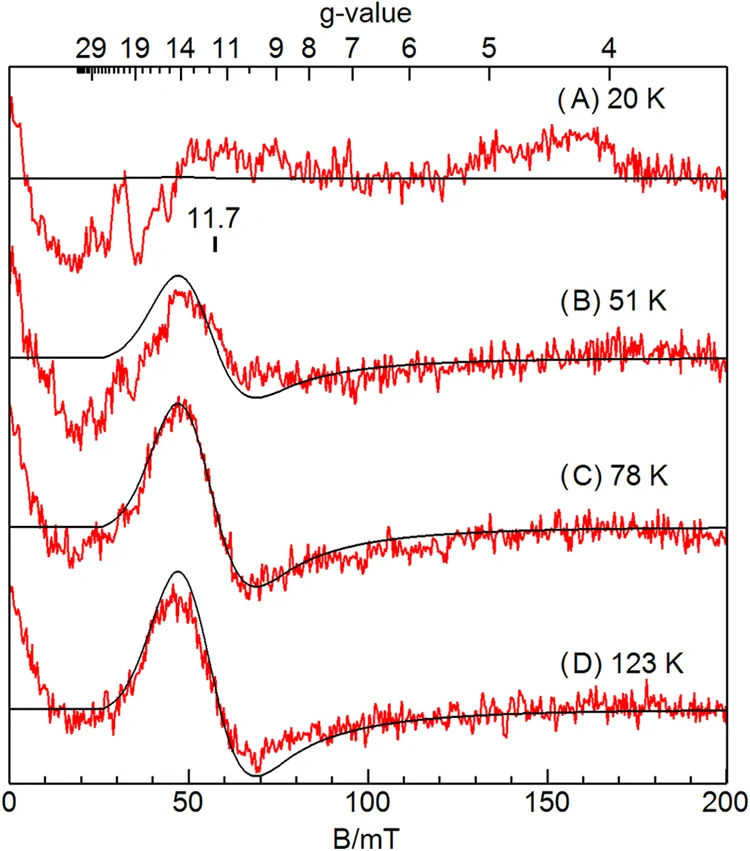
Parallel-mode EPR spectra (red traces, 2 mW at 9.367 GHz) of **7** in PrCN at the temperatures as listed. The black traces are simulations for antiferromagnetically coupled Fe^III^ *S* = 5/2 sites with *J* = 45 cm^–1^.

The Mössbauer spectrum of **7** of the sample prepared at −80 °C was different from that of **5**. The Mössbauer parameters of the [Fe^III^poat] site in **7** were similar to those of the same site in **1**, indicating that the valence and primary coordination sphere at the Fe center of the [poat]^3–^ site were essentially unchanged upon oxidation of the complex. The change in the isomer shift of the other Fe center indicated that oxidation had occurred to afford [Fe^III^TMTACN]. Mössbauer and EPR spectroscopies gave an antiferromagnetic exchange coupling of *J* = 50 cm^–1^ between the two *S* = 5/2 Fe^III^ ions centers. This value is at the upper end of the range expected for monohydroxido bridged complexes and well below that expected for an oxido bridge (*J* ≈ 200 cm^–1^).^8^ As discussed for **1**, the hydrogen bond to the hydroxido bridge raises the value. The value of *J* and the unchanged parameters for the [Fe^III^poat] site indicated that the proton had not yet transferred to [poat]^3–^ and the hydroxido bridge was still present. The optimized DFT structure of **7** gave calculated Mössbauer parameters in agreement with the experiment (Table 1), further supporting its formulation.

### Mössbauer and EPR Properties of [(TMTACN)­Fe^III^(O)­Fe^IV^poat]^2+^ (**8**)

The oxidation of **6** with [N­(*p*-C_6_H_4_Br)_3_]­PF_6_ at −90 °C in PrCN generated complex **8**. An EPR spectrum of **8** at 17 K displayed an isotropic *S* = 1/2 signal at *g* = 2.01.^2^ Spin quantification of the signal gave an amount consistent with the concentration of the complex in solution. Mössbauer measurements of **8** at 4 K and variable magnetic fields gave spectra and simulations (Figure 12) from which Mössbauer parameters of the Fe sites were obtained: δ = 0.00 mm/s, Δ*E*_Q_ = +0.58 mm/s and δ = 0.47 mm/s, Δ*E*_Q_ = −1.11 mm/s, which are typical of *S* = 2 Fe^IV^ and *S* = 5/2 Fe^III^ centers, respectively (Table 1). At higher temperatures in zero magnetic field, the spectrum does not display doublets owing to intermediate spin relaxation. The isomer shift of the Fe^III^ center was close to that of the [Fe^III^TMTACN] sites in the diferric complexes of this work, indicating that the Fe center at the [poat]^3–^ site has been oxidized. The Mössbauer spectra also show a minor diamagnetic diferric species of unknown origin, accounting for 10% of the total iron, which has been included in the simulations of Figure 12. At higher temperatures, the EPR spectra show the same signal with intensity decreasing as a function of 1/T. There is no indication of an excited state and thus a large value of *J* is estimated (>100 cm^–1^, Table 1), which is consistent with the presence of a bridging oxido ligand and the formulation of complex **8**. The gas-phase optimized DFT structure of the oxidized complex gave Fe–O bond lengths consistent with the iron oxidation states (Table S1). Moreover, the Mössbauer parameters obtained from DFT calculations are in good agreement with the experimental values (Table 1), with a calculated *J* value of 150 cm^–1^, suggesting that the DFT structure is in close resemblance to the actual structure.

**Figure 12:**
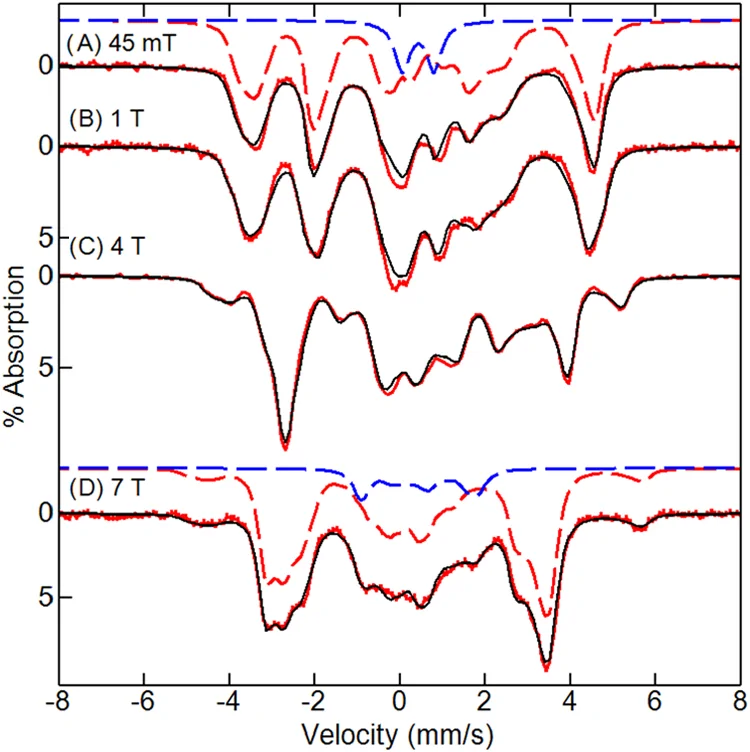
Mössbauer spectra (red traces) of **8** in PrCN recorded at 4.2 K in the magnetic fields as listed. The black traces are global least-squares fits composed of the sum of two species: Fe^III^–O–Fe^IV^ (parameters in Table 1) and an unknown diferric species (δ = 0.44 mm/s, Δ*E*_Q_ = 0.79 mm/s) in a ratio of 90:10. For A and D, the simulation components of the sum are displayed as dashed lines (Fe^III^–O–Fe^IV^, red) and (diferric impurity, blue).

## Discussion

### Correlation of Electronic and Structural Properties

The change in Mössbauer parameters between solution and solid states of the monomeric ferrous complex **2** indicate that the conformations of the complex in the two phases are different. Notably, the sign of Δ*E*_Q_ and the A-tensor changed between the phases without altering the value of the isomer shift (Table 1). Thus, while the electron density at iron nucleus stayed the same, the electron distributions around the iron nucleus differ, indicating a change in the electronic configuration of the iron center.

A change in the sign of the DFT-calculated values for Δ*E*_Q_ was also found between the structure obtained from XRD methods (Figure 4B) and the gas-phase optimized structure (Figure 4A). This finding agrees with the sign change observed in the experimental Δ*E*_Q_ values in the powder versus solution, assuming that the gas-phase DFT structure is a suitable approximation of the molecular structure in the solution. The value of Δ*E*_Q_ is mainly determined by the doubly occupied 3*d* orbital of the *d* ^6^ configuration of the high-spin Fe^II^ metal atom. In the XRD structure of the K_4_[**2**]_2_ cluster, the O=P bonds of the phosphinic amido groups orient toward the two potassium atoms; none of the O=P arms form a H-bond with the Fe–OH moiety. The orientations of the phosphinic amido arms give the doubly occupied 3*d* orbital an oblate character (“xy like”, Figure 4B) and a positive value for Δ*E*_Q_. In the DFT gas-phase structure (Figure 4A), rotations about the N–P bonds cause the O=P bonds of the phosphinic amido groups to point away from the hydroxido ligand bound to the Fe center, preventing any intramolecular H bonds. The same N–P rotation positions one phenyl group of each ligand arm parallel to the Fe–OH unit in a “picket fence” manner (Figure 4A).^11^ The deprotonated N donors carry an electron lone pair directly normal to the C–N–P planes of the phosphinic amido groups (see SI and Figures S9–S11). Rotation of these groups alters the orientation of the N-based lone pairs and the orbital configuration at the Fe center, resulting in a prolate-shaped doubly occupied 3*d* orbital (z^2^ like, Figure 4A) and a negative value for Δ*E*_Q_.

The XRD and DFT structures of **1** show that two of the phosphinic amido arms of [poat]^3–^ provide O-atom donors which, with the hydroxido ligand, complete the primary coordination sphere of the [Fe^II^TMTACN] unit. The O atom of the remaining phosphinic amido arm forms an intramolecular H-bond with the bridging hydroxido ligand. The orientation of the O=P bonds proximal to the Fe^III^–OH unit is similar to that in the molecular structure of {K­[**3**]}_2_, with the Fe center in the [FeTMTACN] fragment occupying a position similar to that of a potassium ion. The same conformations of the O=P units and intramolecular H-bond are also present in the DFT optimized structures of complexes **1** and **4** (Figure 4C). The conformations of the phosphinic amido groups were evident in the spectroscopic data. For both the [Fe^III^poat] and [Fe^II^poat] moieties in complexes **1** and **4**, the ligand conformations and H-bonding interactions caused the values of Δ*E*_Q_ to switch signs relative to that of the mononuclear Fe complexes in solution. The underlying electronic symmetry that causes this sign switch is most evident in **4**, owing to the doubly occupied orbital in [Fe^II^poat], which is an oblate-shaped orbital (Figure 4C), similar to what was found for the solid-state structure of K_4_[**2**]_2_ but with a different orientation.

Overall, the results indicate that the formation of the diiron complexes requires a rotation about the P–N bonds of the phosphinic amido groups in **3** to orient the O=P units for coordination to the [Fe^II^TMTACN]^2+^ moiety (Scheme 1). The existence of similar conformations for both mono-Fe complexes, irrespective of the iron oxidation state, suggests that steric repulsions or π cloud interactions between the phenyl rings in [poat]^3–^ are important in determining the conformation. We suggest below that the change in orientations of the phenyl rings between mono- and di-iron complexes influences the properties of the iron-oxido unit.

**Scheme 1:**
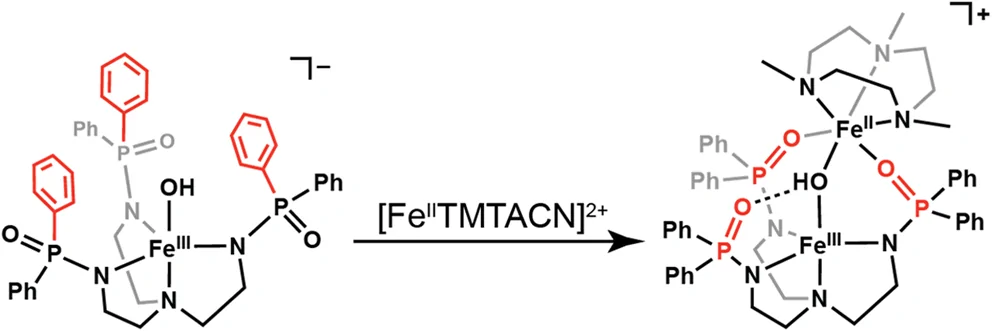
Changes in Conformations of the Phosphinic Amido Groups upon Formation of **1**

### Comparison of the High-Spin Fe^IV^ Site in [(TMTACN)­Fe^III^(O)­Fe^IV^poat]^2+^ (**8**) with Mononuclear Fe^IV^=O Species

Iron­(IV) centers are typically observed in either intermediate (*S*_Fe_ = 1) or high (*S*_Fe_ = 2) spin states. The hyperfine tensor of the [Fe^III^TMTACN] site in **8** is isotropic (Table 1), confirming that the Fe center in [Fe^III^TMTACN] site is high-spin (*S*_Fe_ = 5/2). The EPR spectrum of **8** indicates that the overall spin state is *S* = 1/2, thus the spin state of the [Fe^IV^poat] site must be *S*_Fe_ = 2. Furthermore, the spin-dipolar contribution to the A-tensor (*A*_SD_) gives an indication of the electronic open shell configuration. The experimental *A*_SD_ can be estimated by subtraction of the isotropic value of *A* to give *A*_SD_ = (4, 1, −5) T, assuming that the spin–orbit contributions to *A* are small for Fe^IV^ centers. In agreement with experiment, the DFT calculated *A*_SD_ values for the Fe^IV^ site with an *S*_Fe_ = 2 spin in the broken-symmetry state of **8** are *A*_SD_ = (5, 1, −6) T when expressed in the local *S*_Fe_ = 2 representation. The components of *A*_SD_ for high-spin Fe^IV^–O units are typically axial, with the component with the largest magnitude being twice the magnitude and opposite in sign of the smaller components. For instance, an *A*_SD_ = (4, 4, −8) T was experimentally observed for the mononuclear [Fe^IV^poat­(O)]^−^ complex.^3^ The *A*_SD_ for the Fe^IV^ site of **8** has rhombic symmetry because the Fe–oxido motif is bound to another Fe center with an Fe^IV^–O–Fe^III^ bond angle of 137°. The symmetries of *A*_SD_ reflect the terminal versus bridging nature of the oxido ligand (i.e., Fe^IV^=O vs bent Fe^IV^–O–Fe).

There are now several metalloproteins and synthetic complexes with Mössbauer parameters for *S*_Fe_ = 2 Fe^IV^ centers (Table 2). For complexes in which the terminal oxido ligand has an additional interaction, such as with another Fe center, a main group metal ion, or multiple H-bonds, the Fe–O bond lengths are longer and the oxido ligand donates less electronic density to the Fe center, thereby lowering the isomer shifts. Notice that some of the complexes are supported by anionic N donors whereas others utilize neutral N donors within the equatorial plane; this suggests that the Fe–O bond, rather than the charge of the N donors, is responsible for the observed variations in the isomer shift. For two of the synthetic complexes reported to be 6-coordinate, the isomer shift increases to values greater than 0.2 mm/s. For the Fe^II^ and Fe^III^ complexes in our study, the isomer shift also increases significantly (by approximately 0.15 mm/s) for the 6-coordinate complexes. The greater isomer shifts for the Fe^IV^ intermediates in RNR-*X* and TauD-*J*, which are known to be high-spin, suggests that these complexes are 6-coordinate.

**Table 2:** Mössbauer Parameters of High-Spin (*S*_Fe_ = 2) Fe^IV^ Centers in Synthetic Complexes and Protein Active Sites^a^ ^,^ ^b^

complex	coordination number	Fe–O (Å)	δ (mm/s)	ΔE_Q_ (mm/s)	refs
**8**	5	(1.702)	0.00 (0.00)	+0.53	this work
[Fe^IV^poat(O)---Mg^II^]^+^	5	1.67 (1.665)	0.02 (0.03)	+0.43	^3^
[Fe^IV^H_3_buea(O)]^−^	5	1.680 (1.679)	0.02 (0.03)	+0.43	^12^
[(tpa^Ph^)Fe^IV^(O)]^–^ ^c^	5	1.62	0.09	0.51	^13^
[Fe^IV^(O)(TMG_2_dien)(N_3_)]^+^	5	1.65 (1.649)	0.11 (0.12)	–0.30	^14^
[Fe^IV^(O)(TMG_3_tren)]^2+^	5	1.661 (1.648)	0.09 (0.11)	–0.29	^15^
[Fe^IV^poat(O)]^−^	5	1.65 (1.639)	0.11 (0.12)	+0.27	^3^
[Fe^IV^(O)^t^Bu_3_TACN]^2+^	5	1.65	0.11	0.96	^16^
[Fe^IV^(O)(TQA)(MeCN)]^2+^	6	(1.642)	0.24	–1.05	^17^
Fe^IV^(O)(Me_3_TACN)((OSiPh_2_)_2_O)	6		0.22	+0.23	^18^
RNR-*X*			0.26	–0.6	^19^
TauD-*J*			0.31	–0.88	^20^
[Fe^IV^(O)(H_2_O)_5_]^2+^			0.38	–0.33	^21^

a DFT values in parentheses.

b Ordered by increasing magnitude of their isomer shifts.

c tpa^Ph^ = tris­(5-phenylpyrrol-2-ylmethyl)­amine.

### Proposed Mechanism for Proton Transfer

We have considered mechanisms for the proton transfer (PT) that occurs when **1** is oxidized to **5** (Figure 13). At a reaction temperature of −80 °C, the spectroscopic results presented here indicate that the intermediate species **7** is generated during the process. Upon warming to room temperature **7** converts to **5**, in which the hydroxido ligand is deprotonated with the concomitant protonation of the [poat]^3–^ ligand. This PT event changes the primary coordination sphere around the Fe^III^ center by coordinating the O=P group of the phosphinic amide arm that has been protonated (denoted “unique arm”, Figure 13). The DFT energies of the two di-Fe^III^ structures are nearly degenerate at room temperature, with the hydroxido bridged species marginally lower in energy (Δ*G*_298K_ ≈ 2 kJ/mol or 150 cm^–1^). However, as shown experimentally, **5** is the only observed species in a PrCN solution (Figure 8),^2^ perhaps owing to solvent interactions not included in the DFT calculations or inaccuracies in the DFT energies. A bimolecular PT process involving two di-Fe^III^ complexes is unlikely because of steric repulsions, so our investigations were restricted to intramolecular PT mechanisms. In our previous report, we suggested that the acidic hydroxido proton initially transfers to the phosphinic amido O atom (to which it forms an H-bond), then a tautomerization affords complex **5**.^2^ The analysis of this mechanism is summarized in the SI (Figure S8): the PT from a transient P–OH unit to the adjacent N atom of the phosphinic amide group involves a four-membered ring with an activation energy of ∼200 kJ/mol, which is energetically unfavorable.

**Figure 13:**
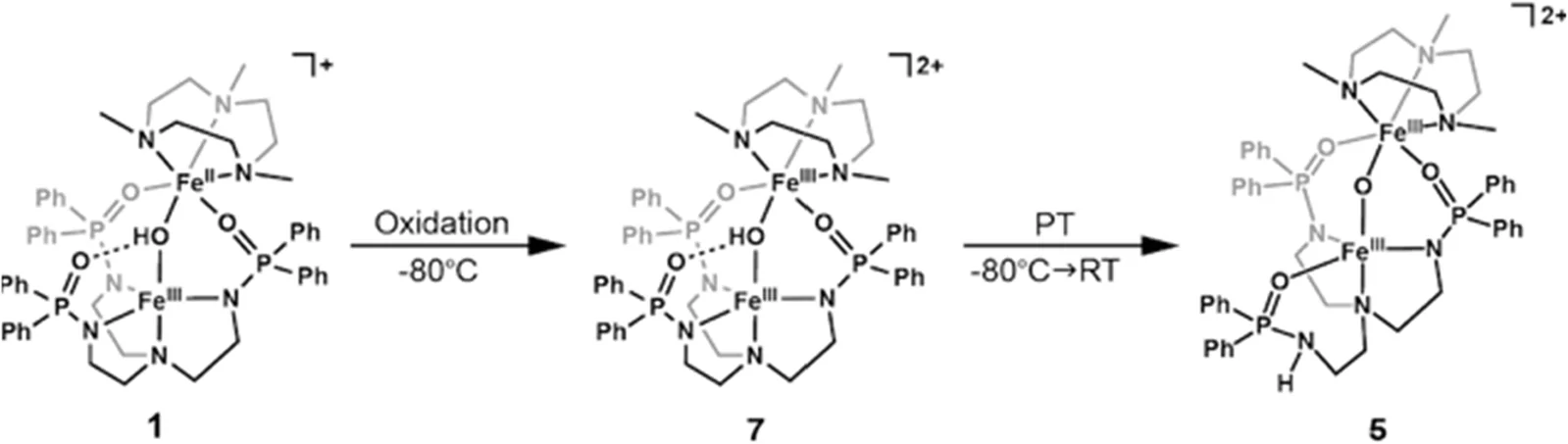
Reaction sequence for conversion of **1** to **5**.

We also examined whether this PT process could be assisted by an external H-bonding source, such as trace water molecules. This proposal is supported by the facile arm rotation observed for the mononuclear species (Figure 4) which would enable binding of an external water molecule (Figure 14, Intermediate 1) via two noncovalent interactions: (1) acting as a H-bond acceptor with the bridging hydroxido group and (2) serving as a H-bond donor with the coordinated N atom. We note that the lone pair on this N atom interacts only marginally with the π system of the adjacent O=P bond (Figures S5–S11), thus facilitating the H-bonding interaction between the incoming water molecule and the coordinated/negatively charged N atom. The two H-bonds to water compensate for the loss of the H-bond between the bridging hydroxido group and the O=P group.

**Figure 14:**
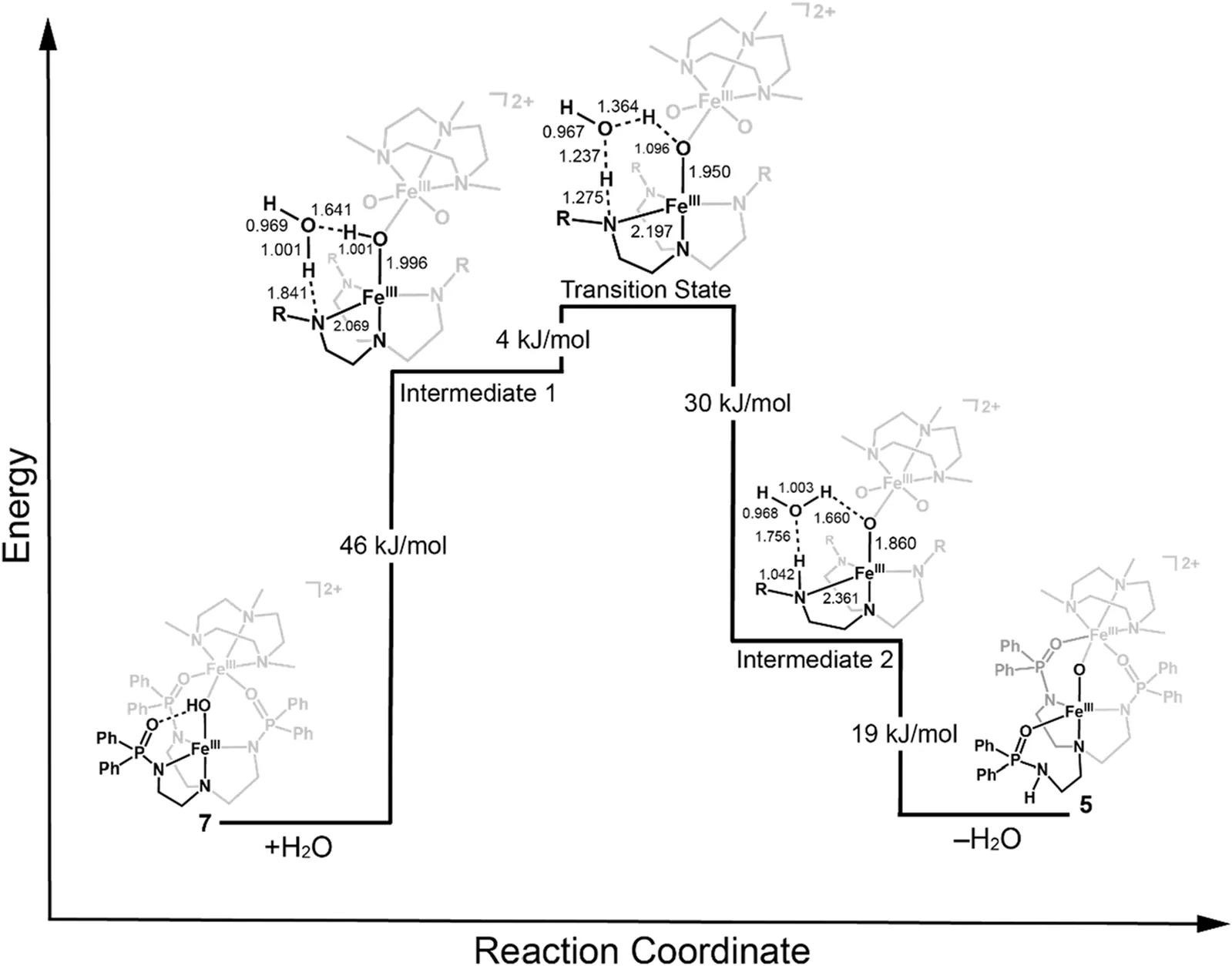
Proposed water-catalyzed proton transfer mechanism for the conversion of complex **7** to complex **5**. Energy level diagram obtained by DFT showing energy differences based on the free energies at 298 K (Δ*G* in kJ/mol). The distances (in Å) affected by the proton transfer are indicated in each intermediate.

After the association of water to complex **7**, the critical concerted H^+^ movement occurs via the six-membered ring formed in intermediate 1 (Figure 14). In addition, the ligand arm involved in the PT has been rotated around the N–Fe bond over an angle of 70° in intermediate 1 relative to initial complex, thereby directing the lone pair at the N atom toward a vacant space favorable to form the H bond with the water molecule. The water molecule promotes a concerted shift of the hydroxido proton to the water and a water proton to the N atom resulting in intermediate 2 (Figure 14), which also contains a H-bond network but one in which the water forms H-bonds with the oxido bridge and the newly formed N–H group. The process passes through a transition state (Figure 14) featuring a hydroxide ion with two H-bonds. The bond distances display the expected trends with a contraction of the (poat)­Fe–O bond length being caused by the formation of an oxido ligand and the increase in the Fe–N bond length being the result of the protonation of the N atom. The weakening of the Fe–N bond in intermediate 2 promotes the full cleavage of the Fe–N bond and the formation of the Fe–O=P bond in tandem with the dissociation of water, leading to formation of complex **5**.

The DFT-calculated energy profile of the proposed water-assisted PT reaction shows the change in free energy at 298 K for each step (Figure 14). The water binding step is endergonic (Δ*G* > 0). The large value calculated for Δ*G* (46 kJ/mol) results from a lowering in translational entropy due to the binding of the water molecule, which is assumed to freely diffuse prior to binding. The activation energy of the intermediate 1 → TS step is small (Δ*G* = 4 kJ/mol) and demonstrates a possible catalytic role of the water molecule. The next step, TS → intermediate 2, is downhill. The final step is water-release and is also downhill because the translational entropy now lowers Δ*G*. The effective free energy of activation of the process is Δ*G* ^⧧^ = 50 kJ/mol (Figure 14) and we have estimated a Δ*G* ^⧧^ range of 33–50 kJ/mol for a spontaneous reaction when the translational motion of the unbound water is either initially bound or roaming freely (see SI after Figure S7).

### Proposed Mechanism for the Reactivity of Complex **8** with Phenols

We have previously reported that **8** reacts at −90̊ C with phenolic substrates to produce **7** and products consistent with the initial formation of phenoxyl radicals; warming to room temperature converts intermediate **7** to **5**.^2^ To gain greater insights into this process, we have explored the mechanism using DFT methods with 2,6-di-*tert-*butyl-phenol as the organic substrate. Computational attempts to transfer an H atom from the phenol directly to the oxido ligand failed because steric repulsions between the di-Fe complex and the substrate inhibited this reaction. Our computational analysis revealed another pathway that involved the unique phosphinic amido arm to facilitate the initial steps in a proton-coupled electron-transfer (PCET) process. Because this unique phosphinic amido group of **8** is not involved in an intramolecular H-bond, it can rotate around the P–N bond, positioning it away from the Fe^III^–(O)–Fe^IV^ core and allowing it to interact with substrates. The XRD structure of **6** and the DFT structure of **8** show this rotational flexibility, but the Fe^III^–(O)–Fe^IV^ core is still substantially shielded by the TMTACN ligand to block direct access to external substrate. However, the flexibility of the unique phosphinic arm suggests it acts as a conduit for a PCET pathway (Figure 15) in which the initial transfer of the phenolic proton to the P=O group occurs with the concurrent transfer of an electron to the Fe^IV^ site.

**Figure 15:**
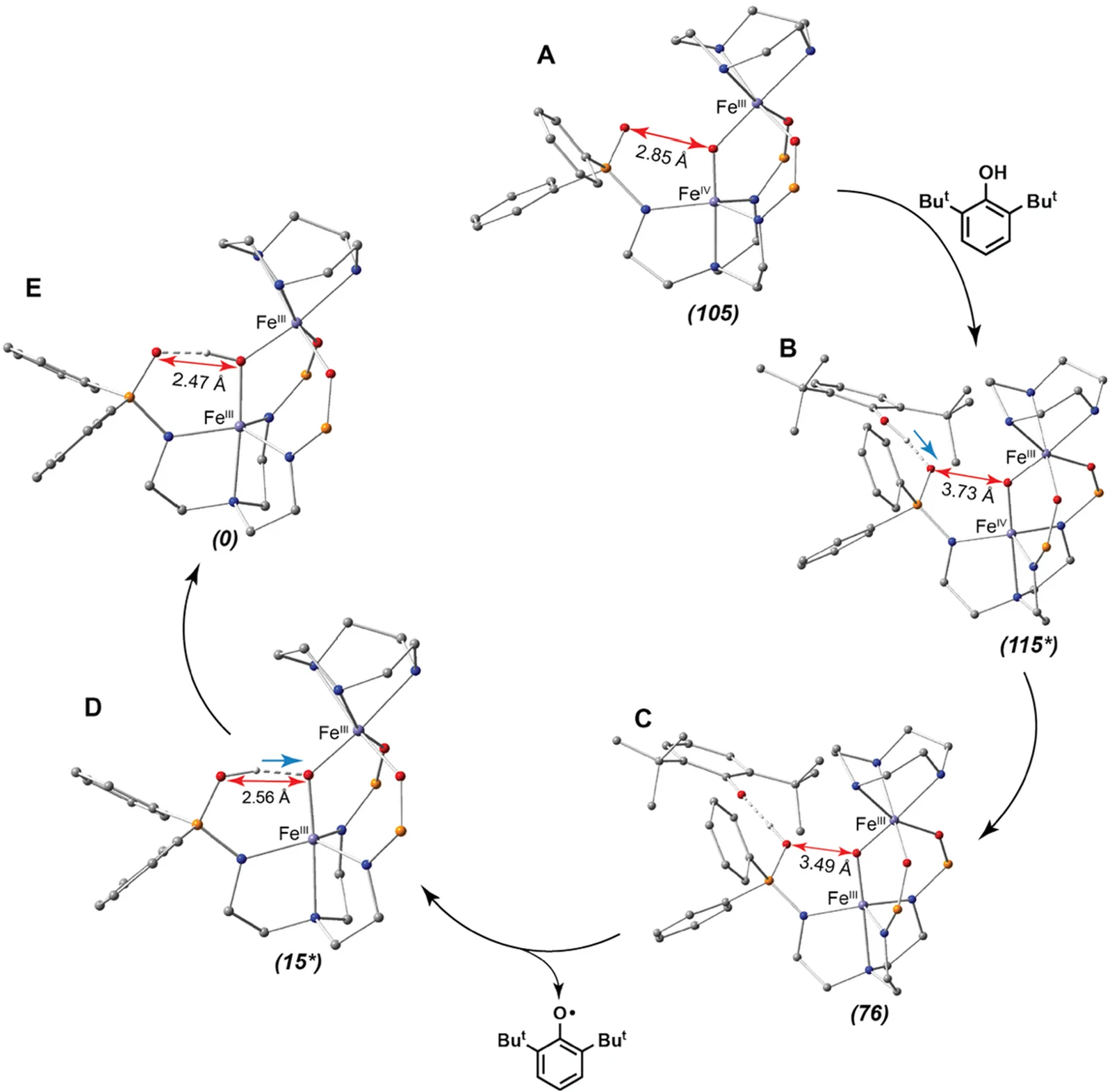
DFT analysis of the H atom abstraction from 2,6-di*^t^* Bu-phenol by complex **8**. Distances denote the changes between the bridging oxido ligand and the P=O group (red double headed arrows). Also highlighted are the key proton transfer steps (blue arrow). The numbers given below each structure are the relative free energies of the DFT structures including the substrate at 298 K (Δ*G*, in kJ/mol). Energies with asterisks are estimates and not from frequency calculations (see SI). For clarity, the methyl groups of TMTACN and the phenyl groups on phosphinic amide groups bonded to the Fe^III^ center are not shown, and only hydrogen atom involved in the PCET mechanism is displayed. Color code: H (white), C (gray), N (blue), O (red), P (yellow), Fe (gray-blue).

From our computations, the first step (Figure 15, A → B) brings the phenolic substrate at H-bonding distance from the unique P=O group in **8**. For the B → C step, the transfer of the phenol proton toward the O=P acceptor occurs (blue arrow) with the concomitant transfer of an electron to the [Fe^IV^poat] site. For the C → D step, the phenoxyl radical dissociates and a rotation of a ligand arm about its N–P bond aligns the P–OH group toward the bridging oxido ligand. In the final D → E step, the subsequent motion of the proton toward the oxido ligand occurs (blue arrow), resulting in the final structure E (complex **7**). The changes in the free energies (Δ*G*) relative to the final complex (E, Figure 15) have been computed and the Δ*G* values have been compared with the corresponding changes in the electronic free energies (Δ*E*_el_, Figure S12). The electronic and free energies are roughly equal for the initial and final state but not for the intermediates where the substrate is associated with the di-Fe complex. The complexation causes a loss of translational entropy, which results in a large positive contribution to Δ*G* and introduces a small barrier (∼10 kJ/mol) in an electronically downhill process.

### Proposed Relevance to RNR

The reaction of **8** with phenols shares structural and functional similarities with the activation of Tyr122 by RNR-*X*, the Fe^III^Fe^IV^ intermediate generated during catalysis (Figure 1).^22–30^ RNR-*X* has a valence-localized Fe^III^–(O)–Fe^IV^ core with two additional bridging carboxylate residues to produce a triply bridged unit; in complex **8** the carboxylate bridges are replaced by the two phosphinic amido bridges. While the ancillary ligands to the Fe centers in RNR-*X* differ from those in **8**, the comparable di-Fe cores prompted us to consider the transformation of Tyr122 to its analogous tyrosyl radical within the context of the PCET mechanism described in the last section. Experimental results indicate that Y122 in RXR-*X* is closer to the Fe^III^ site (Figure 1) than to the Fe^IV^ site and is ∼7 Å from its hydroxyl group, a distance that precludes a direct interaction.^31^ The carboxylate group of Asp84 is coordinated in a κ^1^ manner to the Fe^III^ center and can rotate toward Tyr122 to form a H bond (Figure 1, PDB Code: 1MXR).^31^ We have identified two mechanisms in our complexes that facilitate movement of protons via ligand arm rotations or catalytic water (Figures 14, and 15). These mechanisms may be operative in the RNR mechanism proposed in Figure 1. We suggest that a transient network of hydrogen bonds involving the Asp and Glu arms, and water, provides a through bond interaction between the Tyr and bridging oxido. During the PCET step, the shifting of protons through this network allows protonation of the bridge and reduction of the Fe­(IV) site to produce a metallocofactor with an Fe^III^–(OH)–Fe^III^ core and the Tyr122 radical. Our studies and others have shown that Fe^III^–(OH)–Fe^III^ cores are unstable, converting to the thermodynamically more stable Fe^III^–(O)–Fe^III^ units. In our system, the proton on the bridge in **7** is scavenged by the unique phosphinic amido group to produce **5**.

## Conclusions

The structural dynamism of active sites within metalloproteins is often linked to function and is manifested in a variety of ways. Amino acid residues can act as endogenous ligands to the metal center(s) of a cofactor and often change their binding mode during turnover. Exogenous ligands also have important roles, with water serving as a key ligand which again can readily bind/dissociate during a catalytic process. These primary coordination sphere effects alone cannot account for the reactivities exhibited by metalloproteins: secondary coordination sphere interactions between metallocofactors with endogenous protein groups and/or water molecules are also essential. As we and others have argued, it is the synchronization of the interactions within both coordination-spheres that give biological active sites their unique properties. Unraveling the connections between the primary and secondary coordination spheres and their impact on electronic structure and function is an ongoing pursuit; in metalloproteins these studies are challenging because their inherent catalytic function often prevents detection of key intermediates. It is here that the described work has relevance to electron and proton transfer processes in di-Fe cores found in proteins.

We developed a system which can be sequentially oxidized in one-electron steps from a complex with an Fe^II^–(OH)–Fe^II^ core to its corresponding Fe^IV^–(O)–Fe^III^ species. Each complex was characterized with Mössbauer or EPR spectroscopies for an understanding of electronic structures and determination of exchange couplings. Our spectroscopic findings support structural work that indicated **4** has an intramolecular H-bond between the bridging hydroxido ligand and the P=O unit from one of the phosphinic arms of the [poat]^3–^ ligand (referred to as the unique arm). This arm has been identified as a participant in several subsequent processes involving the higher oxidized constructs. Oxidation of **4** produced **7** which is unstable at room temperature but can be detected at – 80̊ C. Mössbauer spectroscopy showed that the hydroxido ligand remains bridged between the two Fe^III^ centers with *J* = 50 cm^–1^, a value that is in the range found in other Fe^III^–(OH)–Fe^III^ complexes. Moreover, signals were observed in the parallel-mode EPR spectra of **7** that arose from integer coupled-spin states, a finding that is consistent with the lower exchange coupling provided by a hydroxido ligand.

Warming **7** to room temperature afforded tautomer **5**, a complex with an Fe^III^–(O)–Fe^III^ core. Analysis of the Mössbauer spectra gave a *J* value of greater than 100 cm^–1^, a value typically found in di-Fe^III^ complexes in which antiferromagnetic coupling is mediated by a bridging oxido ligand. The OH^–^ → O^2–^ conversion causes a pronounced structural change at the Fe^III^ center within the [poat]^3–^ ligand whereby the proton is transferred to the N atom of the unique arm. As a result, the primary coordinate sphere of the Fe^III^ center is altered, as the P=O group is now bound, causing a distortion in the trigonal plane and a commensurate increase in isomer shift. Evaluating this PT process using DFT suggests a mechanism involving the initial binding of a water molecule to **7**. The water is contained within the secondary coordination sphere and is positioned between the hydroxido ligand and the unique arm (intermediate 1, Figure 14) via H-bonds. The water is thus part of a transient H-bonding network and serves as a conduit to facilitate protonation. Similar types of protonation processes that utilize water molecules are often proposed in the active site of proteins (e.g., deprotonation processes in carbonic anhydrase, and proton transfer from H_2_O_2_ in peroxidases).^32,33^ However, we are not aware that it has been invoked for the deprotonation of synthetic complexes with Fe^III^–(OH)–Fe^III^ cores, possibly because of the limited amount of ligands that are capable of forming H-bonds.

Complex **5** can be deprotonated to afford **6**, another complex with an Fe^III^–(O)–Fe^III^ core but one with all the N atoms of [poat]^3–^ coordinated to the Fe^III^ ion. A major structural rearrangement ensued upon deprotonation with the trigonal plane in **6** reverting to those found in **1** and **7**. The structural similarities around the [Fe^III^poat] fragments in all three complexes is supported by their nearly identical Mössbauer parameters for this Fe^III^ center (Table 1). The isolation of **6** allowed us to form **8**, a complex with an Fe^III^–(O)–Fe^IV^ core: our Mössbauer and EPR studies support that the Fe^IV^ center is bound to the [poat]^3–^ ligand and it is high spin. However, the parameters found for **8** do not match those found in the related di-Fe cofactor RNR-*X*, which could be caused by differences in the type and number of ligands coordinated to the Fe^IV^ center.

Previously, it was shown that **8** reacts with external phenols via homolytic cleavage of their O–H bond and a PCET process was proposed to initially form **7** and phenoxy radicals. This process was further probed using DFT methods and the unique arm of [poat]^3–^ was again found to have an important mechanistic role. There is no intramolecular H-bond in **8** (nor **5** and **6**), leading to its phosphinic amido group of the unique arm to be untethered. Therefore, the P=O group can rotate such that an *intermolecular* H-bond can be formed with the hydroxyl group of the phenolic substrate (Figure 15B). This interaction provides a pathway to transfer the electron to the Fe^IV^ center and the proton to the oxido, forming **7**. The premise that a ligand on an Fe^III^–(O)–Fe^IV^ core can shuttle protons/electrons via H-bonds may have biological relevance, especially in RNR where a carboxylate group that is coordinate to the di-Fe cofactor could rotate and interact with the tyrosine residue in a similar manner to what is proposed in **8** (Figures 1 and 15). Probing these types of processes is possible with synthetic systems through the development of multifunctional ligands such as [poat]^3–^, which can support high spin Fe centers, serve as a synthon to form discrete bimetallic complexes, and promote both intra- and intermolecular H-bonds to facilitate proton/electron transfer steps.

## Experimental Section

All reagents were purchased from commercial sources and used as received unless otherwise noted. Solvents were sparged with argon and purified using a JC Meyer Co. solvent purification system with columns containing Q-5 and molecular sieves. Potassium hydride (KH) as a 30% dispersion in mineral oil was filtered with a medium porosity glass frit and washed 5 times each with pentane and diethyl ether (Et_2_O). Solid KH was dried under vacuum and stored under an inert atmosphere. All synthetic manipulations were conducted in a Vacuum Atmosphere, Co. drybox under an argon atmosphere. All complexes for Mössbauer spectroscopy in this study are ^57^Fe-enriched. The ^57^Fe-enriched complexes were prepared using ^57^Fe^II^(OAc)_2_ and ^57^Fe^II^(OTf)_2_·2CH_3_CN, which were synthesized according to procedures for the natural abundance Fe precursors.^34,35^ Elemental analyses were performed on a PerkinElmer 2400 Series II CHNS elemental analyzer.

### Preparative Methods

The preparative routes for all the diiron complexes and the potassium salt of [Fe^III^poat­(OH)]^−^ (**3**) have been reported;^6^ the synthesis for the new salt, K_2_[Fe^II^poat­(OH)] (K_2_[**2**]), is described below.

#### Preparation of K_2_[**2**]

To a solution of H_3_poat (0.200 g, 0.268 mmol) in anhydrous THF (6 mL) was added KH (0.033 g, 0.82 mmol), and the reaction was allowed to proceed until gas evolution ceased and all solids were dissolved. To the solution was added Fe^II^(OAc)_2_ (0.048 g, 0.28 mmol). The yellow solution was stirred for 25 min, and then H_2_O (5 μL, 0.3 mmol) was added via syringe. After 5 min, the reaction mixture was filtered through a medium fritted glass funnel to remove insoluble material. KH (0.011 g, 0.27 mmol) was added to the filtrate, which turned orange and was allowed to stir for 10 min. The reaction mixture was then filtered through a medium fritted glass funnel to remove any insoluble material. Orange crystals (86%) were afforded by vapor diffusion of diethyl ether (Et_2_O) into the THF filtrate. Elemental analysis calcd. (found) for K_2_[Fe^II^poat­(OH)]·THF, C_42_H_43_FeK_2_N_4_O_4_P_3_·C_4_H_8_O; C, 57.14 (57.03); H, 5.32 (5.67); N, 5.79 (5.54) %. FTIR (ATR, cm^–1^): 3631, 3049, 2944, 2821, 1481, 1433, 1277, 1243, 1176, 1168, 1155, 1146, 1114, 1046, 1027, 961, 925, 746, 696, 620, 608, 583, 579, 575, 568, 561, 558, 554. X-ray diffraction information is detailed in the SI.

### X-ray Crystallographic Methods

K_4_[**2**]_2_·4THF. A Bruker SMART APEX II diffractometer was used to collect all data. The APEX2 program package was used to determine the unit cell parameters and for data collection.^36^ The raw frame data were processed using SAINT and SADABS to yield the reflective data files. Subsequent calculations were carried out using the SHELXTL program.^37^ The structure was solved by direct methods and refined on F2 by full-matrix least-squares techniques. The analytical scattering factor7 for neutral atoms were used throughout the analysis. Hydrogen atom H(1) was located from the difference-Fourier map and refined (x,y,z and U_iso_). The remaining hydrogen atoms were included using a riding model. The molecule was located about an inversion center.

### Spectroscopic Methods

Solid-state Fourier transform infrared (FTIR) spectra were collected on a Thermo Scientific Nicolet iS5 FT-IR spectrometer equipped with an iD5 ATR accessory. Electron paramagnetic resonance (EPR) spectra were recorded on a Bruker ELEXSYS spectrometer equipped with an Oxford ESR-910 liquid helium cryostat and a Bruker bimodal cavity for the generation of microwave fields parallel (parallel mode) and transverse to the applied magnetic field. The quantification of all signals was performed relative to a CuEDTA spin standard prepared from a copper atomic absorption standard (Sigma-Aldrich). The microwave frequency was calibrated with a frequency counter, and the magnetic field was calibrated with an NMR gaussmeter. The sample temperature was calibrated against a calibrated cernox sensor (Lakeshore CX-1050) mounted inside an EPR tube. A modulation frequency of 100 kHz was used for all EPR spectra. Mössbauer spectra were recorded on either a variable-field or a weak-field spectrometer operating in a constant acceleration mode in a transmission geometry using Janis Research Inc. cryostats that allow for a variation in temperature from 4 to 300 K. One of the dewars housed a superconducting magnet that allowed for the application of magnetic fields up to 8 T parallel to the γ-radiation. Isomer shifts are reported relative to Fe metal at 298 K. The simulation software written by one of the authors (SpinCount)^38^ diagonalizes the electronic terms of the spin Hamiltonian

$$H=JS_{1}\cdot S_{2}+\Sigma_{i=1,2}\left[\beta S_{i}\cdot g_{i}\cdot B+S_{i}\cdot D_{i}\cdot S_{i}+S_{i}\cdot A_{i}\cdot I_{i}-g_{n}\beta_{n}B\cdot I_{i}+I_{i}\cdot P_{i}\cdot I_{i}\right]$$

$$S_{i}\cdot D_{i}\cdot S_{i}=D_{i}\left[{S_{i,z}}^{2}-S_{i}\left(S_{i}+1\right)/3+\left(E_{i}/D_{i}\right)\left({S_{i,x}}^{2}-{S_{i,y}}^{2}\right)\right]$$

$$I_{i}\cdot P_{i}\cdot I_{i}=\left({\mathrm{eQV}}_{i,\mathrm{zz}}/12\right)\left[3I_{i,z}^{2}-I\left(I+1\right)+\eta_{i}\left(I_{i,x}^{2}-I_{i,y}^{2}\right)\right]$$

where the parameters have the usual definitions and perform least-squares fitting of simulations to the spectra. The quantitative simulations are generated with consideration of all intensity factors, which allows the computation of simulated spectra for a specified sample concentration.

### DFT Methods

The DFT calculations were performed with *Gaussian ’09*,^39^ using the hybrid functional B3LYP. The exchange coupling parameters of the Fe^m+^Fe^m′+^ dimers were calculated with the expression *J* = 2­(*E*_F_ – *E*_BS_)/*n*_1_ *n*_2_ where *n*_1_ and *n*_2_ are the number of unpaired electrons on ions Fe^m+^ and Fe^m’+^, respectively, and *E*_F_ and *E*_BS_ are the energies for the optimized geometries of the ferromagnetic (F) and the broken symmetry (BS) configuration. This expression for *J* follows from the expression *J* = 2­(*E*_F_ – *E*_BS_)/(⟨ ***S*** ^2^⟩_F_ – ⟨ ***S*** ^2^⟩_BS_)_DFT_ by replacing the denominator with the product *n*_1_ *n*_2_. This approximation is accurate within 0.6%. In our previous work, satisfactory *J* values were obtained with functional B3LYP, using basis set 6–311G for nonheme diiron complexes. We replaced 6–311G basis with the Triple Zeta Valence Polarization (TZVP) basis set for the P atoms to describe penta-valency of these atoms. The *J* value calculations for the diferrous state required a careful inspection of the orbital configuration to ascertain that the broken symmetry state and ferromagnetic state have the same 3d orbital ground configuration. The calculations for the ^57^Fe isomer shifts (δ) and quadrupole splitting (Δ*E*_Q_) for the Fe^II^ sites were calibrated using a set of previously characterized ferrous model complexes. The isomer shifts for Fe^III^ and Fe^IV^ were calibrated using a training set of Fe^III^, Fe^IV^, and Fe^V^ complexes supported by macrocyclic equatorial ligands and oxido, chloro, aqua, or acetonitrile as axial ligands.

## Supplementary Material



## Notes

Additional structural information and Supporting Figures; additional comments concerning: Influence of hydrogen bonding on *J*, proton transfer mechanism, H atom abstraction mechanism, phosphinic amido group of the [poat]^3–^ ligand; Mössbauer spectrum of K_2_[**2**] powder (PDF)

## §

Department of Chemistry, College of the Holey Cross, 1 College Street, Worcester, Massachusetts 01610, United States
