# Supplementary material for: Redox-Triggered Proton Transfer in Synthetic Models for Non-Heme Diiron Enzymes: Spectroscopic, Computational, and Mechanistic Insights
Source: Inorg Chem. 2026 Sep 25;65(40):24067–81. doi: 10.1021/acs.inorgchem.6c04733 (PMC13664081; doi:10.1021/acs.inorgchem.6c04733)
Supplement: Supplementary file 1 [file ic6c04733_si_001.pdf]

Supporting Information

Redox-Triggered Proton Transfer in Synthetic Models for Non-Heme Diiron Enzymes: Spectroscopic, Computational, and Mechanistic Insights

Justin L. Lee,[‡] Saborni Biswas,[⊥] Victoria F. Oswald,[‡] Joseph W. Ziller,[‡] A.S. Borovik,[‡]* Emile L. Bominaar,[⊥]* Michael P. Hendrich,[⊥]*

[⊥]Department of Chemistry, Department of Chemistry, Carnegie Mellon University, 4400 Fifth Avenue, Pittsburgh, PA 15213

[‡]Department of Chemistry, 1102 Natural Sciences II, University of California, Irvine, CA 92697

Corresponding Author: Michael P. Hendrich, hendrich@andrew.cmu.edu

Table of Contents

Structure of $K_4[\mathbf{2}]_2$•4THF S2

Fe-O distances from DFT S2

DFT conformations of **2** S3

Influence of hydrogen bonding on *J* S5

$[Fe^{II}poat]^{-}$ moiety in **4** S7

$[Fe^{III}(poat)]$ moiety in **3** and **1** S8

Intramolecular proton transfer mechanism S9

The phosphinic amido groups of the $[poat]^{3-}$ ligand S11

H-atom abstraction mechanism S15

Mössbauer quadrupole splittings for the Fe^{II} ions of **4** as a function of temperature. S16

Mössbauer spectra of $K_2[\mathbf{2}]$ in powder S17

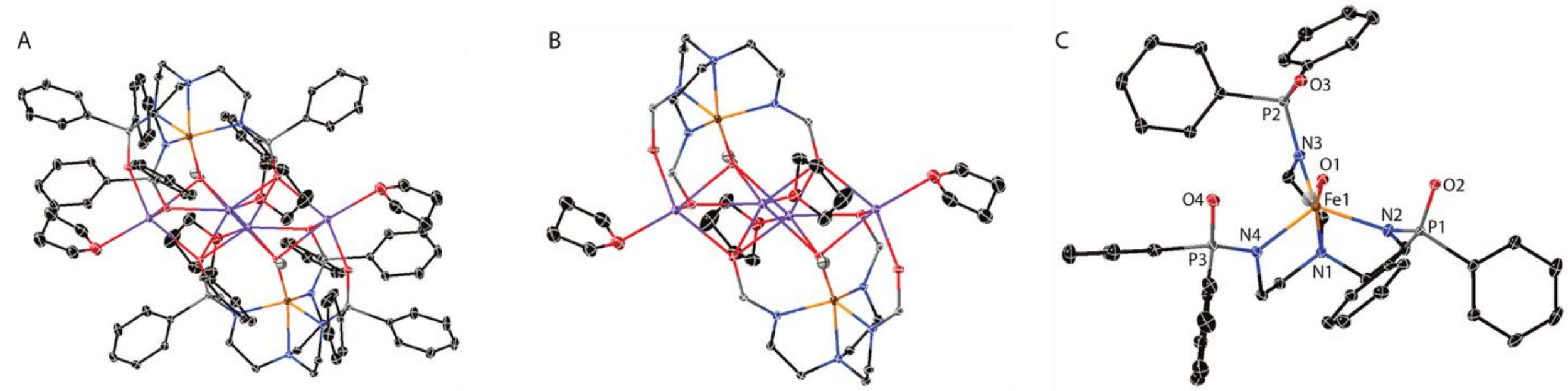

Figure S1. Thermal ellipsoid diagrams depicting the molecular structures of the $K_4[\mathbf{2}]_2$•4THF cluster (A), the cluster with phenyl rings from the [poat]$^{3-}$ ligands removed, and the [**2**]$^{2-}$ fragment. Ellipsoids are drawn at the 50 % probability level, and only the hydroxido H atoms are shown for clarity. Selected bond lengths (Å) and angles (°): Fe1–O1, 2.032(1), Fe1–N1, 2.344(1), Fe1–N2, 2.141(1), Fe1–N3, 2.122(1), Fe1–N4, 2.121(1); O1–Fe1–N1, 165.36(3), N2–Fe1–N3, 116.28(4), N2–Fe1–N4, 124.05(4), N3–Fe1–N4, 106.46(4).

Table S1. Fe-O distances from DFT.

#	Complex	(TMTACN)Fe-O (Å)	O-Fe(H)poat (Å)
**1**	[(TMTACN)Fe^{II}(OH)Fe^{III}poat]$^{+}$	2.017	1.888
**2**$_{solution}$	[Fe^{II}poat(OH)]$^{2–}$		2.002
**2**$_{solid}$	K_2[Fe^{II}poat(OH)]		2.031
**3**	[Fe^{III}poat(OH)]$^{–}$		1.872
**4**	[(TMTACN)Fe^{II}(OH)Fe^{II}poat]	1.924	2.041
**5**	[(TMTACN)Fe^{III}(O)Fe^{III}Hpoat]$^{2+}$	1.811	1.833
**6**	[(TMTACN)Fe^{III}(O)Fe^{III}poat]$^{+}$	1.786	1.830
**7**	[(TMTACN)Fe^{III}(OH)Fe^{III}poat]$^{2+}$	1.896	1.954
**8**	[(TMTACN)Fe^{III}(O)Fe^{IV}poat]$^{2+}$	1.877	1.702

[Fe^{II}poat(OH)]$^{2-}$ complex (2)

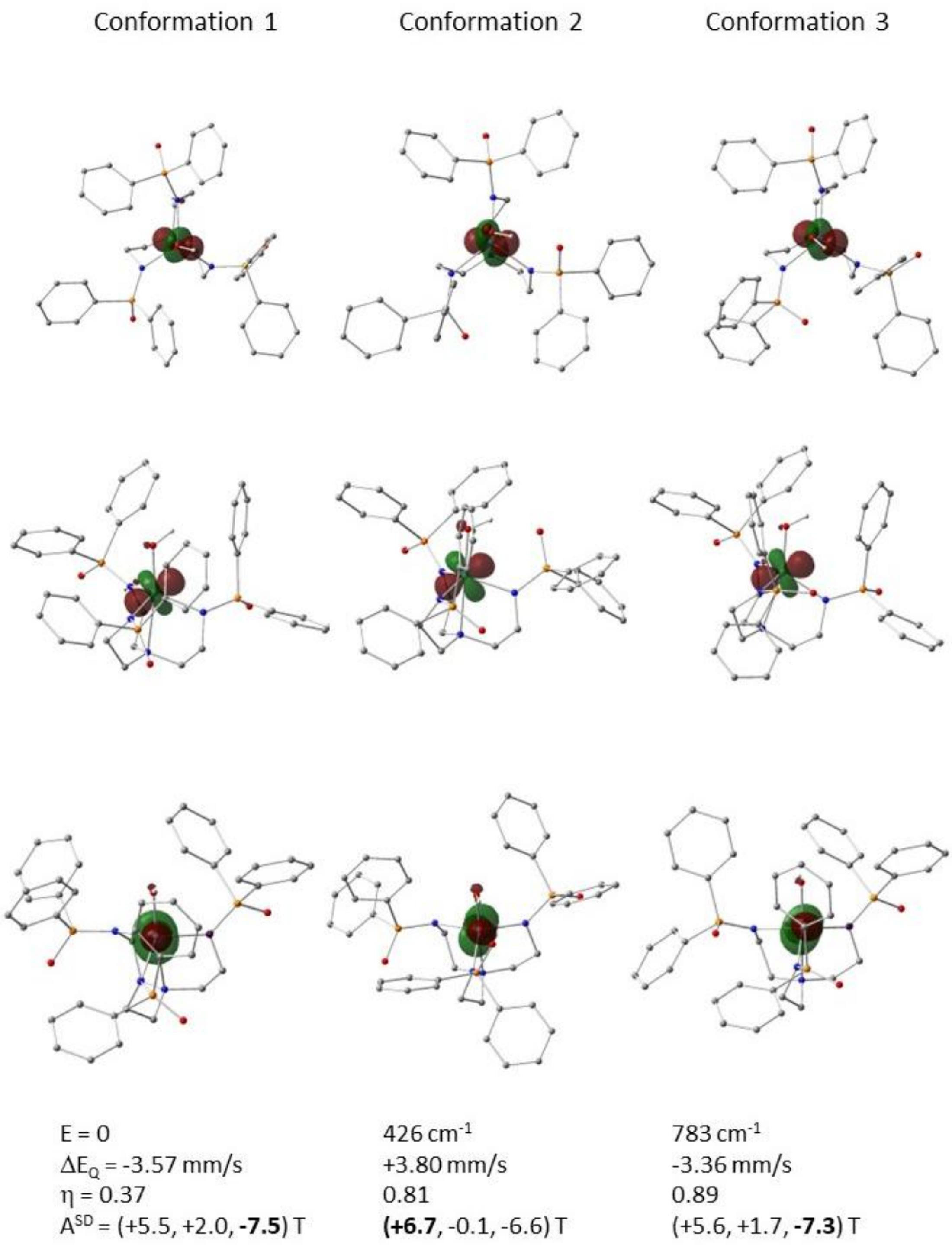

Figure S2. Contour plots of the doubly occupied 3d orbital in three DFT gas-phase optimized conformations of the d^6 high-spin [Fe^{II}poat(OH)]$^{2-}$ complex (**2**) viewed from three perspectives (top, middle, bottom). The relative energies, ^{57}Fe quadrupole splitting parameters and spin-dipolar couplings (in the local S_{Fe} = 2 representation) are given for each conformation. For clarity, the H atoms, apart from the one on the bridge, are not shown. Color code: C (grey), N (blue), O (red), P (yellow), and Fe (blue grey).

The bulkiness of the side chains of the $[poat]^{3-}$ ligand gives rise to steric interactions that lead to rotational barriers of these groups and its constituents, the phenyl rings. As a result, the complex has several stable conformations, which give stationary solutions in DFT geometry optimizations. The optimized geometries for three of these solutions are presented in Figure S2, together with their relative energies and the DFT values for the quadrupole splitting, asymmetry parameter, and spin-dipolar couplings. The lowest energy conformation, labeled 1, has $OP(Ph)_2$ groups in which the O=P vector approximately coincides with the line of intersection of the planes defined by the two phenyl groups, which is the lowest energy conformation for an isolated arm (see below). The other two conformations, which are a few hundred wavenumbers higher in energy, have at least one $OP(Ph)_2$ group in which the O=P vector lies well outside one of the phenyl planes.

None of the P=O oxygen atoms engages in hydrogen bonding with the hydroxido ligand in any of the gas-phase optimized conformations of Figure S2, even when the geometry optimization was initiated using a structure in which such a bond was present. The hyperfine parameters ΔE_Q, η, and $\mathbf{A}^{SD}$ are mainly determined by the nature of the doubly occupied 3d orbital of the d^6 configuration of the high-spin Fe^{II} metal atom. The contour of this orbital has been shown in Figure S2 from three different perspectives (top, middle, bottom). In all three conformations, labeled 1, 2, and 3, the doubly occupied 3d orbital resembles a dz^2 orbital with coordinate z referring to an intermediate spatial direction that does not align with any of the five metal-ligand bonds (see Figure S2).

Among the three conformations, the purest manifestation of a dz^2 orbital is found for conformation 1, leading to a small value for η and negative values for both ΔE_Q and the component of $\mathbf{A}^{SD}$ with the largest magnitude, A^{SD}_{max}. In conformations 2 and 3, the doubly occupied 3d orbital has acquired some dz^2-x^2 character (z roughly along Fe-O_H), causing an increase in the values of η and in the case of conformation 2 a sign flip in both ΔE_Q and A^{SD}_{max}. The best agreement with experimental hyperfine data for the complex in solution is obtained for the lowest energy conformation 1. The negative signs deduced from experiment for ΔE_Q and A^{SD}_{max} reflect the predominant dz^2 character of the doubly occupied 3d orbital. The DFT values for the hyperfine parameters of conformation 1 have been listed in parentheses in Table 1 of the main text. The gas-phase optimized structures of Figure S2 differ from the XRD structures of the $[Fe^{II}poat(OH)]^{2-}$ moiety in complexes in which this moiety is bound to a K^+-crown ether or $[Fe^{II}(TMTACN)]^{2+}$. In these binuclear species, the bridging hydroxido group and two of the P=O groups of the $[poat]^{3-}$ ligand form coordination bonds to an external metal (K or Fe), while the third P=O group accepts a hydrogen bond from the hydroxido bridge, thereby roughly aligning all three P=O bonds with the Fe-O_H bond. As we shall discuss below, the conformational change of the $[Fe^{II}poat(OH)]^{2-}$ moiety induced by the formation of these dimeric species has a profound effect on its electronic structure.

Influence of hydrogen bonding on *J*

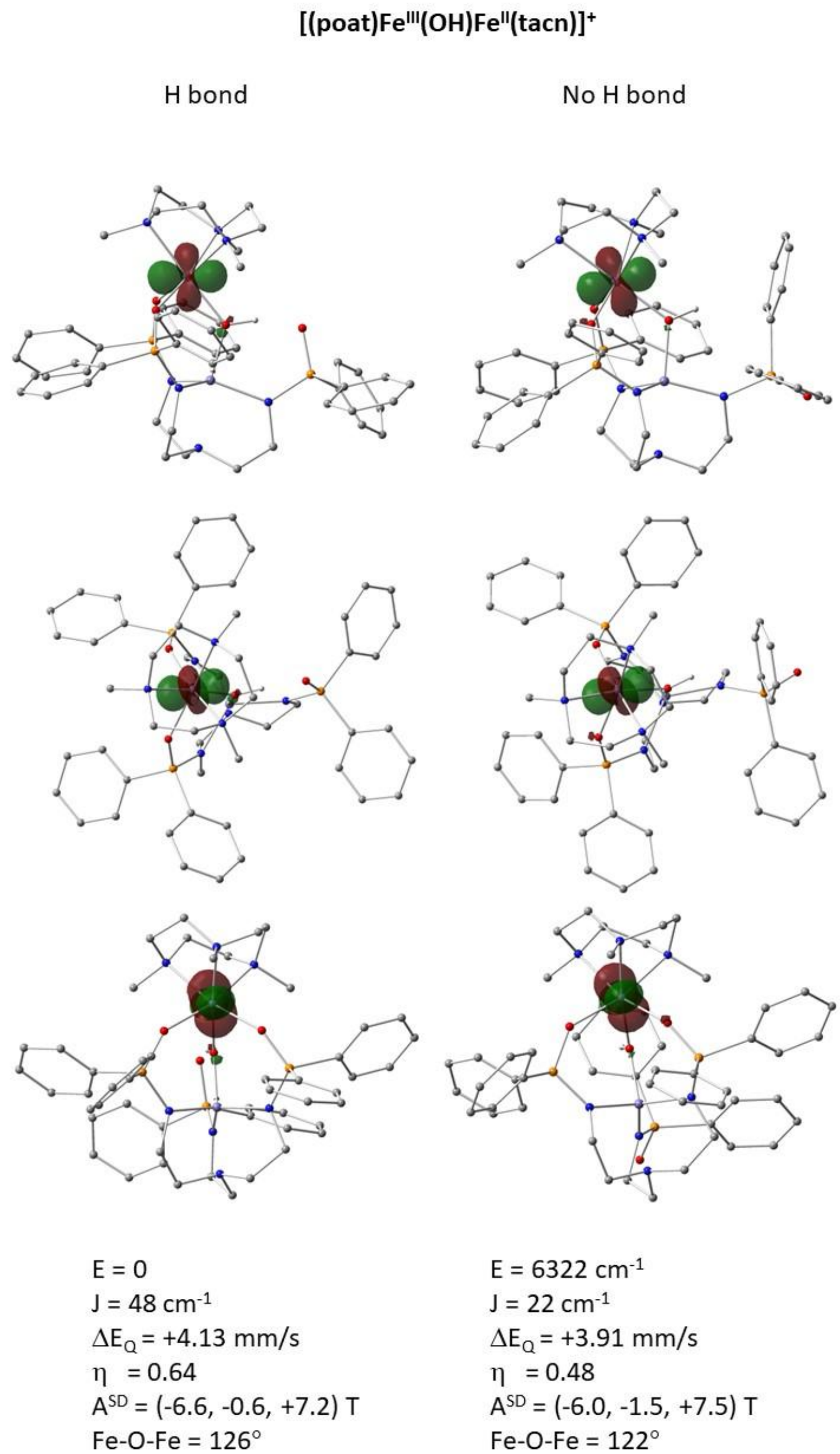

Figure S3. Contour plots of the doubly occupied 3d orbital at the [Fe^{II}(TMTACN)]$^{2+}$ site in two broken symmetry DFT gas-phase optimized conformations of the [(TMTACN)Fe^{II}(OH)Fe^{III}poat]$^+$ complex (**1**), one in which the bridging hydroxido ligand is H-bonded to the O=P group in one of the [poat]$^{3-}$ arms (left) and the other in which there is no such H-bond due to a rotation of one of the [poat]$^{3-}$ arms (right), are shown from three perspectives (top, middle, bottom). The relative energy, *J* values, ^{57}Fe quadrupole splitting parameters, spin-dipolar couplings for the Fe^{II} site (in local S_{Fe} = 2 representation), and bridging bond angle are given for each conformation. For clarity, the H atoms, apart from the one on the bridge, are not shown. Color code: C (grey), N (blue), O (red), P (yellow), and Fe (blue grey).

Figure S3 shows the DFT optimized structures for two conformations of the [(TMTACN)Fe^{II}(OH)Fe^{III}poat]$^+$ cationic complex (**1**), one in which the bridging hydroxido ligand donates a hydrogen bond to the P=O oxygen atom in one of the ligand arms of the [poat]$^{3-}$ ligand and the other one lacking a hydrogen bond due to a rotation of the ligand arm. The figure also displays the contour plots of the doubly occupied 3d orbital in the d^6 configuration of the [Fe^{II}(TMTACN)]$^{2+}$ moiety for the two conformations. Comparing the two contours and their associated hyperfine parameters reveals that the doubly occupied 3d orbitals in the two conformations are similar. The d^5 orbital configurations of the high-spin [Fe^{III}poat] moieties in the two conformations are equal. Thus, even though in both cases a hydroxido bridge couples irons with similar electronic states, the associated exchange-coupling constants (*J*) obtained from DFT differ by more than a factor of two (see Figure S3). The largest *J* value arises for the conformation in which the hydroxido bridge is hydrogen bonded, suggesting that this interaction has increased the calculated value for *J*. The influence of hydrogen bonding on *J* can be explained by noting this interaction weakens the O–H bond in the hydroxido bridge, thereby conferring some oxido character onto the hydroxido ligand, resulting in a larger value for *J*. The larger value of 48 cm^{-1} compares favorably with the experimental value of 40 cm^{-1} .

Electronic structure of the [Fe^{II}poat]$^{–}$ moiety in the binuclear species [(TMTACN)Fe^{II}(OH)Fe^{II}poat] (4).

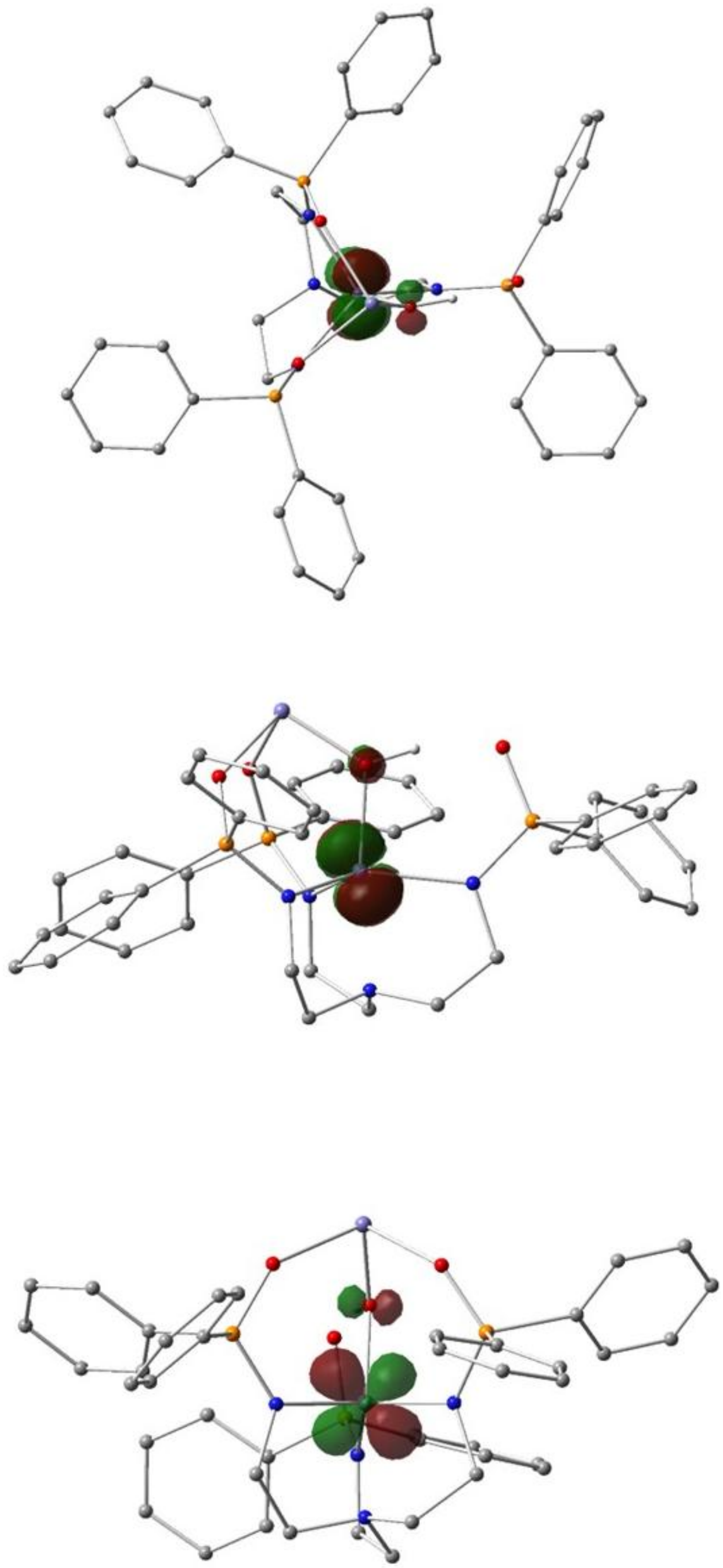

Figure S4. Contour plot of the doubly occupied 3d orbital of the d^{6} high-spin [Fe^{II}(poat)]$^{1-}$ moiety in [(TMTACN)Fe^{II}(OH)Fe^{II}poat] (**4**) is shown from three perspectives in the DFT optimized structure. For clarity, the TMTACN ligand and H atoms, apart from the one on the hydroxido bridge, are not shown. Color code: C (grey), N (blue), O (red), P (yellow), and Fe (blue grey).

Figure S4 shows the DFT optimized structure of the $[Fe^{II}(poat)]^{2+}$ moiety in the $[(TMTACN)Fe^{II}(OH)Fe^{II}poat]$ complex (**4**) from three perspectives. The figure reveals that the P=O bonds are approximately aligned with the (poat)Fe-O_H bond and that one P=O accepts a hydrogen bond from the hydroxido bridge while the other two P=O are coordinated to the TMTACN-bound iron. Figure S4 also displays the contour plot of the doubly occupied 3d orbital at the $[poat]^{3-}$-bound Fe site as obtained by DFT. Comparison with Figure S2 shows that the orbital in the dimer complex (**4**) is different from those in the conformations 1, 2, and 3 of the monomer complex (**2**). The doubly occupied 3d orbital in the dimeric species has dxz character, with the z axis being tilted from the Fe-O_H bond by about 30° and the x axis defined normal to the O-H···O=P plane containing the hydrogen bond. Unambiguously, the orbital in the dimer gives positive values for both ΔE_Q and the component of the $\mathbf{A}^{SD}$ tensor with the largest magnitude, $A^{SD}{}_{max}$, in agreement with the signs deduced from experiment. The conformational change of the $[poat]^{3-}$ ligand, induced by the formation of the dimeric species, has caused a significant change in the orbital structure of the $[Fe^{II}poat]^{-}$ moiety, leading to a sign flip in both ΔE_Q and $A^{SD}{}_{max}$.

$[Fe^{III}(poat)]$ site in $[Fe^{III}poat(OH)]^{-}$ (3) and $[(TMTACN)Fe^{II}(OH)Fe^{III}poat]^{+}$ (1)

A comparison of the DFT gas-phase structures of the high-spin $[Fe^{III}poat(OH)]^{-}$ complex anion and the $[(TMTACN)Fe^{II}(OH)Fe^{III}poat]^{+}$ cationic dinuclear species shows that the $[poat]^{3-}$ ligand undergoes a similar conformational change as occurs between the gas-phase monomeric and dimeric Fe^{II} species described above. Thus, in the dinuclear structures the P=O vectors are roughly aligned with the Fe-O_H bond but rotate in the gas-phase structure of mononuclear $[Fe^{III}poat(OH)]^{-}$ similar as in the monomeric Fe^{II} species. The Fe^{III} version of conformation 1 has again the lowest energy, while conformation 3 is 557 cm^{-1} higher in energy. Finding similar low-energy conformations irrespective of the iron oxidation state demonstrates the predominant role of the steric interactions between the side chains of the $[poat]^{3-}$ ligand in defining the conformation. Ideally, the spherical half-filled 3d shell of high-spin Fe^{III} gives $\Delta E_Q = 0$ and $\mathbf{A}^{SD} = 0$. However, anisotropic donations of electron density by the ligand into the empty 3d orbitals result in small but non-vanishing values for these quantities. The DFT values for $\Delta E_Q = + 0.64$ mm/s and $\eta = 0.41$ for the ferric version of the lowest energy conformation 1 have been listed in parentheses in Table 1. The values for the ferric version of conformation 3 are $\Delta E_Q = -0.74$ mm/s and $\eta = 0.79$, indicating a sign change of ΔE_Q compared to conformation 1. Thus, like for the $[Fe^{II}poat(OH)]^{2-}$ fragment, the DFT analysis of the $[Fe^{III}poat(OH)]^{-}$ fragment shows that ΔE_Q for this Fe^{III} site depends on the $[poat]^{3-}$ conformation as well. However, the modifications in anisotropic covalency underlying these changes are intricate and will not be further pursued here. DFT calculations of the dinuclear complex $[(TMTACN)Fe^{II}(OH)Fe^{III}poat]^{+}$, in which the P=O groups are roughly aligned with the (poat)Fe-O_H bond, gives for the $[Fe^{III}poat]$ moiety the values $\Delta E_Q = -0.68$ mm/s and $\eta = 0.24$ (Table 1), indicating a sign flip of ΔE_Q compared to the value of this parameter for the lowest energy conformation of mononuclear $[Fe^{III}poat(OH)]^{-}$ in the gas phase.

Comments on intramolecular proton transfer mechanism

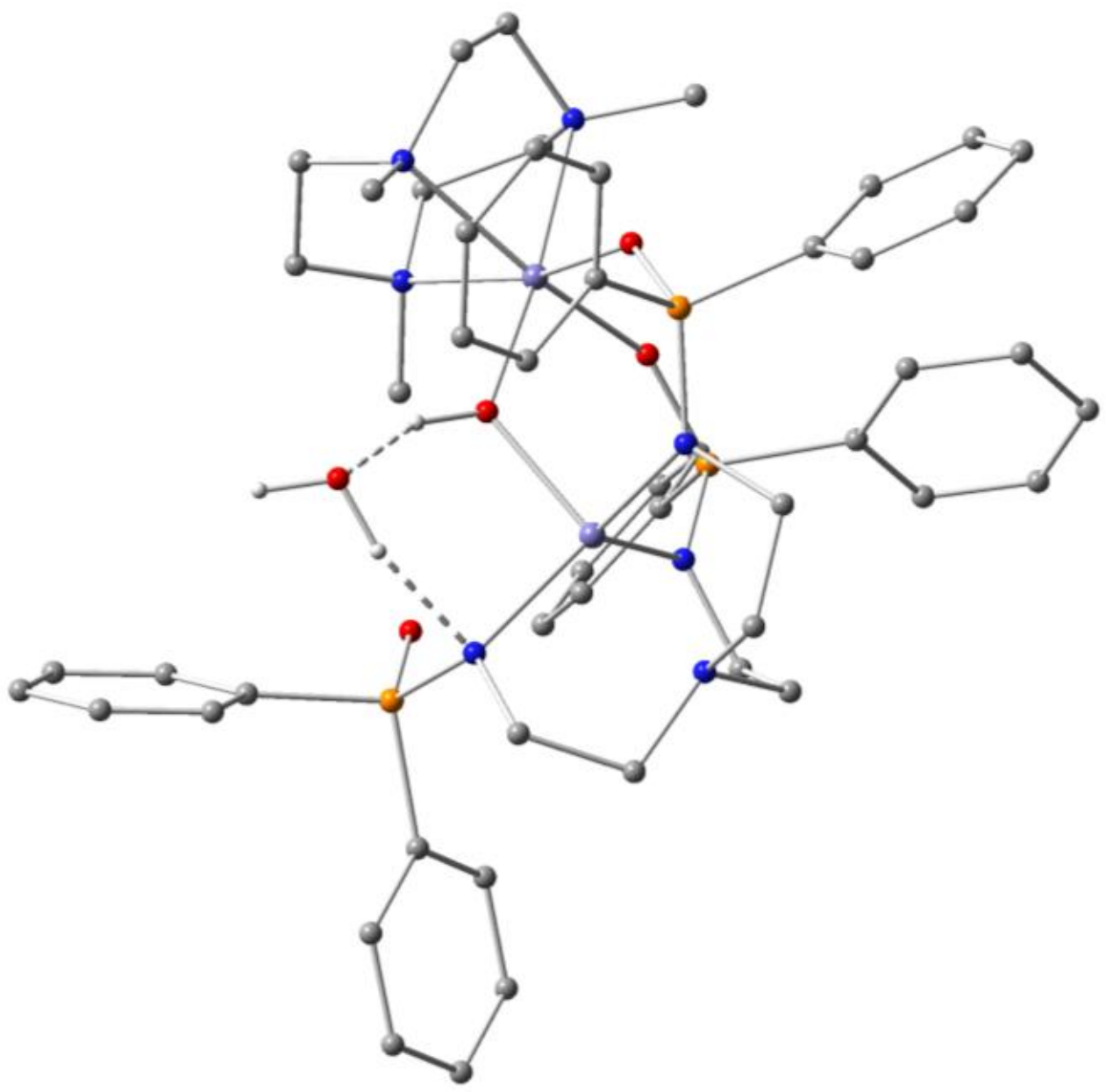

Figure S5. DFT structure of the water-bound hydroxido-bridged/[poat]$^{3-}$ intermediate species, Int1.

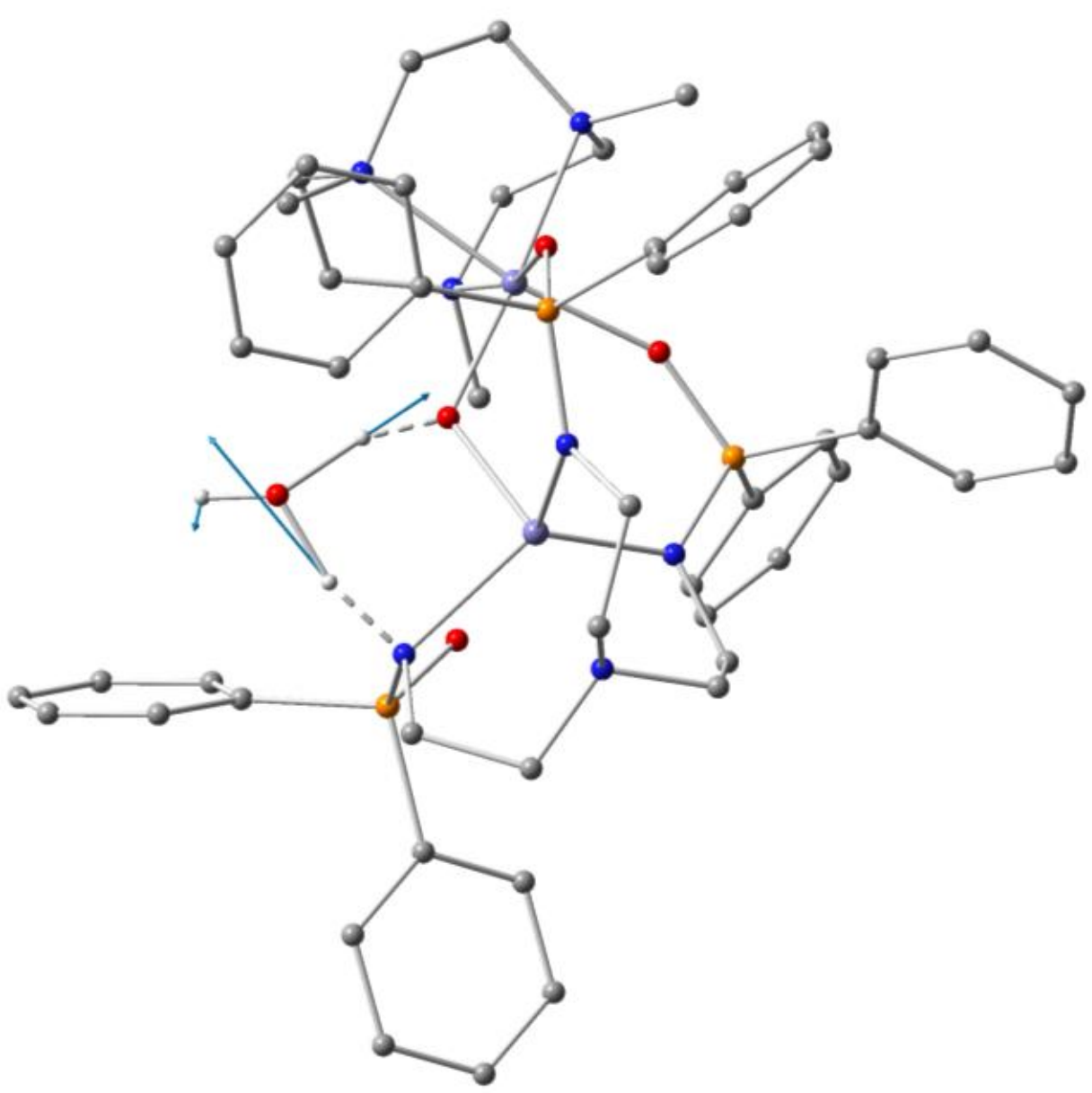

Figure S6. DFT structure of the transition state (TS) between the water bound intermediates Int1 and Int2. The blue arrows indicate the atomic displacement vectors of the imaginary frequency mode with $-ih\nu$ = 1010 cm^{-1} and show the collective motion of the protons that give rise to the proton transfer reaction Int1 $\rightarrow$ Int2.

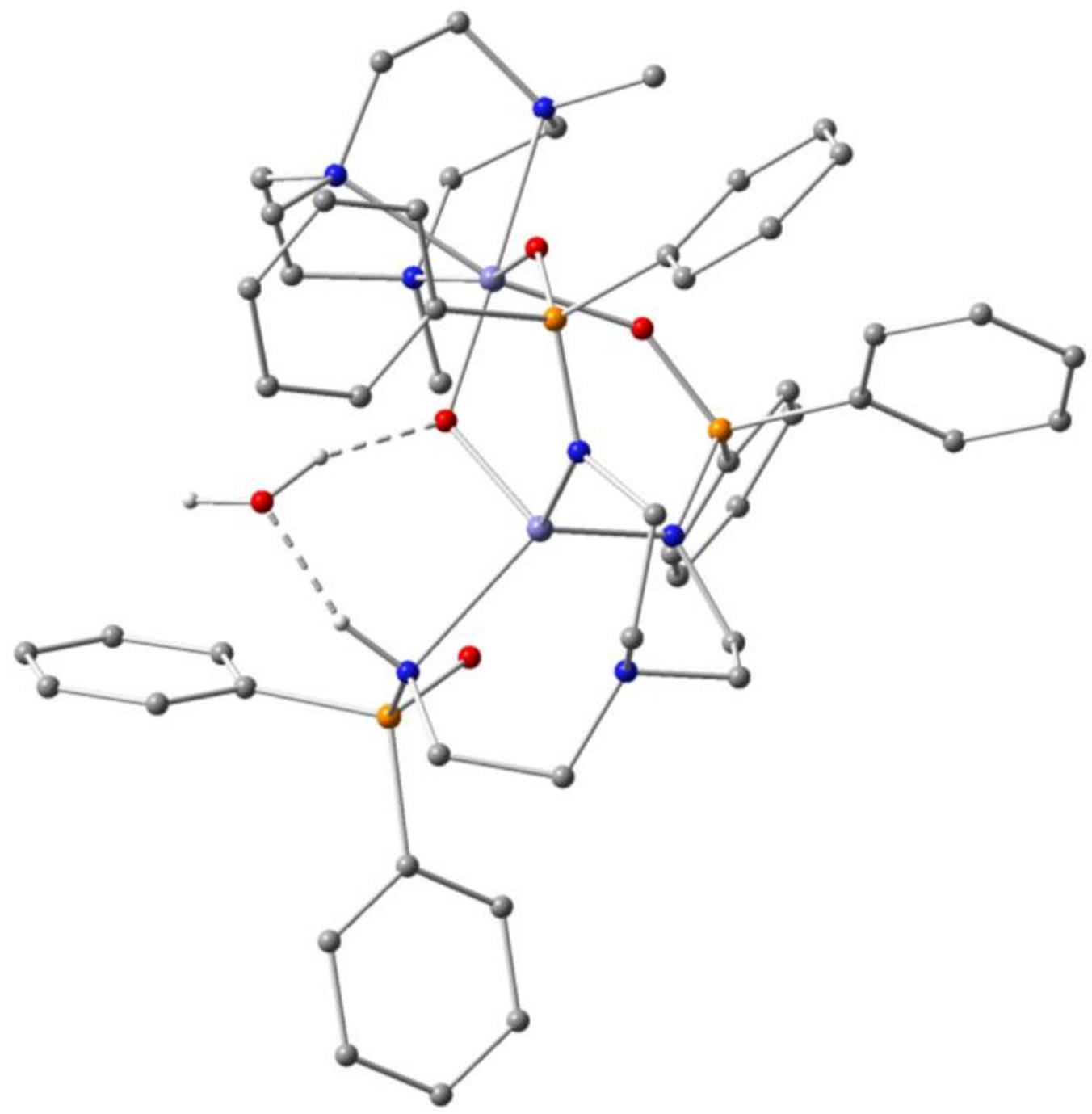

Figure S7. DFT structure of the water-bound oxido-bridged/$[Hpoat]^{2-}$ intermediate species, Int2.

Estimate of the activation free energy for the transformation initial state + water $\rightarrow$ TS, accounting for a variable degree of translational freedom of the catalytic water. Denoting the free energies for water binding to the OH-bridged complex and water release from the Hpoat complex as ($\Delta G_{binding}$, $\Delta G_{release}$), we obtained the values (46, –17) kJ/mol in the case that the translational motion of the water prior to binding is unencumbered (estimated using DFT values for ΔG at 298 K) and (0, 29) kJ/mol when the translational motion of the unbound water is fully constrained (estimated using DFT values for E_{el} + E_{ZPE}). In the intermediate case where the translational water motion is partially constrained, the expression is (46 – Δ, –17 + Δ) kJ/mol with Δ = 0 for the free case and Δ = 46 kJ/mol for the fully constrained case. The condition for spontaneous water release, $\Delta G_{release} < 0$, gives the condition $\Delta < 17$ kJ/mol, implying that $\Delta G_{binding} > 29$ kJ/mol. The latter quantity, together with the free energy change for Int1 $\rightarrow$ TS (~ 4 kJ/mol), gives a range 33 kJ/mol < ΔG^* < 50 kJ/mol for the effective activation free energy barrier at 298 K.

Intra-molecular proton transfer unassisted by water.

The four diagrams in Figure S8 illustrate the energetics of the intra-molecular transfer of the proton on the hydroxido bridge of the di-Fe^{III} species to the iron coordinating N atom in the arm of the $[poat]^{3-}$ ligand that is not coordinated to the $[Fe^{III}(TMTACN)]^{3+}$ *without* assistance of a water molecule. The top-left structure is the optimized geometry of the hydroxido-bridged di-Fe^{III} species. When the proton of the hydroxido bridge is moved away from O_{bridge}, the proton is first captured by the proximal P=O oxygen. The optimized geometry for a fixed H···O_{bridge} distance of 1.45 Å is shown top-right. The P–OH group in the latter structure is H bonded to O_{bridge}. The distance of the proton to the N acceptor atom remains large as in the top-left structure (~ 2.7 Å). The energy penalty of the proton movement is modest (0.1 eV). When

the proton is moved closer to the N acceptor atom, the proton remains bonded to the P=O group while its distance to O_{bridge} increases. The bottom-left cartoon shows the optimized structure for a fixed H···N distance of ~ 1.8 Å. The H···O_{bridge} distance in this structure has increased to 1.8 Å. Meanwhile, the Fe–N distance has only slightly lengthened, but the total energy has increased by the sizable amount of 1.4 eV. Further shortening of the H···N distance further raises the energy. The bottom-right cartoon shows the optimized structure for a fixed H···N distance of 1.5 Å. Although the Fe–N bond is further activated, the energy has increased by 2 eV relative to the energy of the initial structure, suggesting that the transition state must be even higher in energy. Eventually, the energy drops again to near zero when the H–N bond is formed. These calculations suggest an activation energy > 2 eV, which is prohibitive and motivated the search for an alternative mechanism for the intramolecular proton transfer.

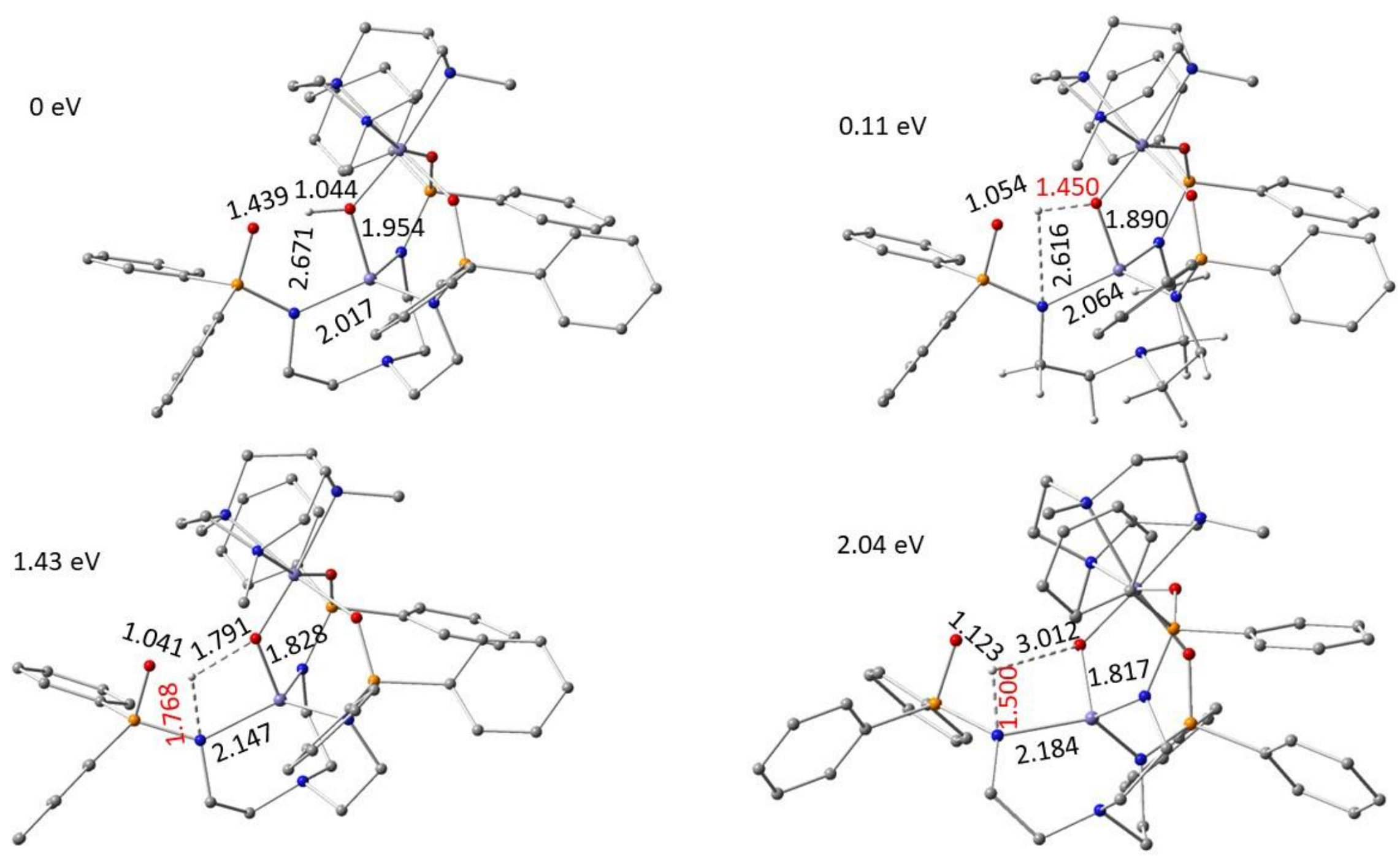

Figure S8. Top left: DFT optimized structure of the [(TMTACN)Fe^{III}(OH)Fe^{III}poat]$^{2+}$ complex (**7**). The other three structures have been optimized using the constrained distances (in Å) indicated in red color and represent intermediate stages of a direct transfer of the proton on the hydroxido bridge to the N of the [poat]$^{3-}$ arm that is hydrogen bonded to the bridging hydroxido group. Other key distances are indicated in black. All energies are relative to the top-left structure.

The phosphinic amido groups of the [poat]$^{3–}$ ligand: Their structure, electronic state, and interaction with iron.

In this section, we discuss whether: (i) the N atoms of the phosphinic amido groups in the [poat]$^{3-}$ ligand carry a π lone pair, (ii) the OPNC unit is planar, and (iii) Fe is inside or outside the CNP plane of an [poat]$^{3-}$ arm.

(i) The proposed mechanism for the intramolecular proton transfer in the [(TMTACN)Fe^{III}(OH)Fe^{III}poat]$^{2+}$ complex (**7**) relied on the notion that the iron-coordinating N atoms of the [poat]$^{3-}$ ligand carry two lone

pairs each: one pair that is accommodated by a hybrid sp^2 orbital forming a coordinative σ bond to iron, the other one by a π orbital available for receiving a proton. In carbonic amido groups [O=C(R)NR–], which are planar, the partial π bonding between C and N compromises the ability of the π electrons to form new bonds. However, the situation differs in phosphinic amido groups ($[O=P(Ph)_2NR]^-$), because the P atom therein is penta-valent, such that there is no unambiguous π-orbital-defining nodal plane like in carbonic amido groups. To identify the intrinsic properties of the phosphinic amido groups in the $[poat]^{3-}$ ligand, DFT calculations were performed on the model $O=P(Ph)_2NH_2$ and its deprotonated form $[O=P(Ph)_2NH]^-$, the latter mimicking the $[poat]^{3-}$ arms in the $[Fe^{m+}poat]^{n+}$ complexes. The optimized structures are shown in Figures S9 and S10 with superimposed the contour plots of occupied π type orbitals with significant amplitudes at the N atom. Figures S9 and S10 show that there is no π bonding between N and P (zero amplitude at P) in either the protonated or deprotonated form. Figure S9 shows a significant mixing of the N-based π orbital with an O-based orbital for the protonated case. Hence, engaging the π orbital at N in the formation of a bond to an incoming proton would require disruption of its interaction with the O-based orbital thereby raising the activation energy of the proton transfer. However, the situation changes in the deprotonated case. The π orbital shown in Figure S10 is the HOMO and almost exclusively localized at the N atom, unlike in the protonated case shown in Figure S9, where the corresponding N-based π orbital appears as a component of two lower lying orbitals (see caption of Figure S9). The lone pair π orbital in the deprotonated case, which applies to the $[poat]^{3-}$ arms in $[Fe^{m+}poat]^{n+}$ complexes, is available for accepting a proton without first having to disrupt a pre-existing bond.

(ii) The OCNC' unit in carbonic amido groups is planar but the OPNC unit in phosphinic amido groups is not. A measure for the non-planarity is the dihedral O-P-N-C angle. DFT values for this angle have been listed in Tables S2 and S3 for the mono- and binuclear $[poat]^{3-}$ complexes $[Fe^{II}poat(OH)]^{2-}$ and $[(TMTACN)Fe^{II}(OH)Fe^{II}poat]$, respectively, and those for the corresponding dihedral O-P-N-H angles in the fragment models $O=P(Ph)_2NH_2$ and $[O=P(Ph)_2NH]^-$ are given in Tables S4 and S5, respectively. The dihedral angles O-P-N-H/H' of $\pm 63^\circ$ (Table S4) and O-P-N-H of -59° (Table S5) have nearly equal values and show that the OPNH moiety is non-planar in both the protonated and deprotonated model system. The O-P-N-C angle in two of the three arms of the mononuclear complex is close to the corresponding O-P-N-H/H' angles in $O=P(Ph)_2NH_2$ and $[O=P(Ph)_2NH]^-$. In contrast, in the binuclear complex, the O-P-N-C angles are all close to 180°, indicative of nearly planar OPNC units. Figure S11 shows the DFT structure of $[O=P(Ph)_2NH]^-$ optimized under the constraint that O-P-N-H is fixed to 180° with superimposed the contour plot of the HOMO, which has retained the features of a lone pair π orbital localized at the N atom. Comparison with the contour plot of Figure S10 shows that the lone pair π orbital has followed the rotation of H around the N–P axis. The energy of the constrained structure in Figure S11 is 1896 cm^{-1} higher than the energy of the fully optimized structure in Figure S10 (using b3lyp/tzvp). The increase in the conformational energies in the $[poat]^{3-}$ arms of the dinuclear species is compensated by the coordination of the P=O groups to the TMTACN-bound Fe in two of the ligand arms and by hydrogen bonding of the P=O group to the hydroxido bridge in the third arm. The rearrangement of the $[poat]^{3-}$ arms in passing from the mononuclear to the dinuclear species is further illustrated by the changes in the dihedral O-P···Fe-$O_{axial/bridge}$ angle from approximately anti-aligned (Table S2) to aligned (Table S3).

(iii) A measure for the position of iron relative to the CNP planes of the $[poat]^{3-}$ arms is the dihedral angle Fe-N-C-P. The Fe-N-C-P angles for the three $[poat]^{3-}$ arms are close to 180° in both the mononuclear and dinuclear complexes (see Tables S2 and S3) and place the iron near the CNP planes of the $[poat]^{3-}$ arms in

both systems. (In contrast, H lies well outside the H'NP plane in $O{=}P(Ph)_2NH_2$.) The CNP plane coincides with the nodal plane of the N-based lone pair π orbital (see Figure S10 and S11). The lone pair π orbitals of the $[poat]^{3-}$ arms interact with matching dπ orbitals of iron. These interactions are of stereochemical significance. The π–dπ overlap is optimal when the iron is placed in the nodal plane of the lone pair π orbital, which may explain why the iron is located near the CNP planes of the $[poat]^{3-}$ arms. The rotation of the lone pair π orbital in Figure S11 (resembling a $[poat]^{3-}$ arm in the dinuclear species) relative to the lone pair orbital in Figure S10 (resembling a $[poat]^{3-}$ arm in the mononuclear species in solution) may have affected the doubly occupied 3d orbital of the $[poat]^{3-}$-bound iron, flipping the sign of quadrupole splitting.

Table S2. Dihedrals in degrees for the three phosphinic amido groups in $[Fe^{II}poat(OH)]^{2-}$ from DFT optimization in gas phase.

Fe-N-C-P [a]	O-P-N-C [b]	O-P···Fe-O_{axial} [c]
161	66	–120
164	57	–151
178	22	–176

[a] 0° or 180° for Fe in the PNC plane.
[b] 0° or 180° for planar OPNC unit (like in carbonic amido groups).
[c] 0° for O=P aligned with Fe–O_{axial} and 180° for O=P anti-aligned with Fe–O_{axial}.

Table S3. Dihedrals in degrees for three phosphinic amido groups in $[(TMTACN)Fe^{II}(OH)Fe^{II}poat]$ from DFT optimization.

Fe-N-C-P [a]	O-P-N-C [b]	O-P···Fe-O_{bridge} [c]
175	172	–13
178	160	–28
–170	–163	34

[a] 0° or 180° for Fe in the PNC plane.
[b] 0° or 180° for planar OPNC unit (like those in carbonic amido groups).
[c] 0° for O=P aligned with Fe–O_{bridge} and 180° for O=P anti-aligned with Fe–O_{bridge}.

Table S4. Dihedrals in degrees in the computational model $O{=}P(Ph)_2NH_2$ from DFT optimization.

H-N-H'-P [a]	O-P-N-H/H' [b]
–129	63/–63

[a] 0° or 180° for H in the PNH' plane, H/H' is corresponding to Fe/C in $[poat]^{3-}$ complexes.
[b] 0° or 180° for planar OPNH/H' units (like those of carbonic amido groups).

Table S5. Dihedrals in degrees in the computational model $[OP(Ph)_2NH]^-$ from DFT optimization.

O-P-N-H [a]
–57

[a] 0° or 180° for planar OPNH units (like those of carbonic amido groups).

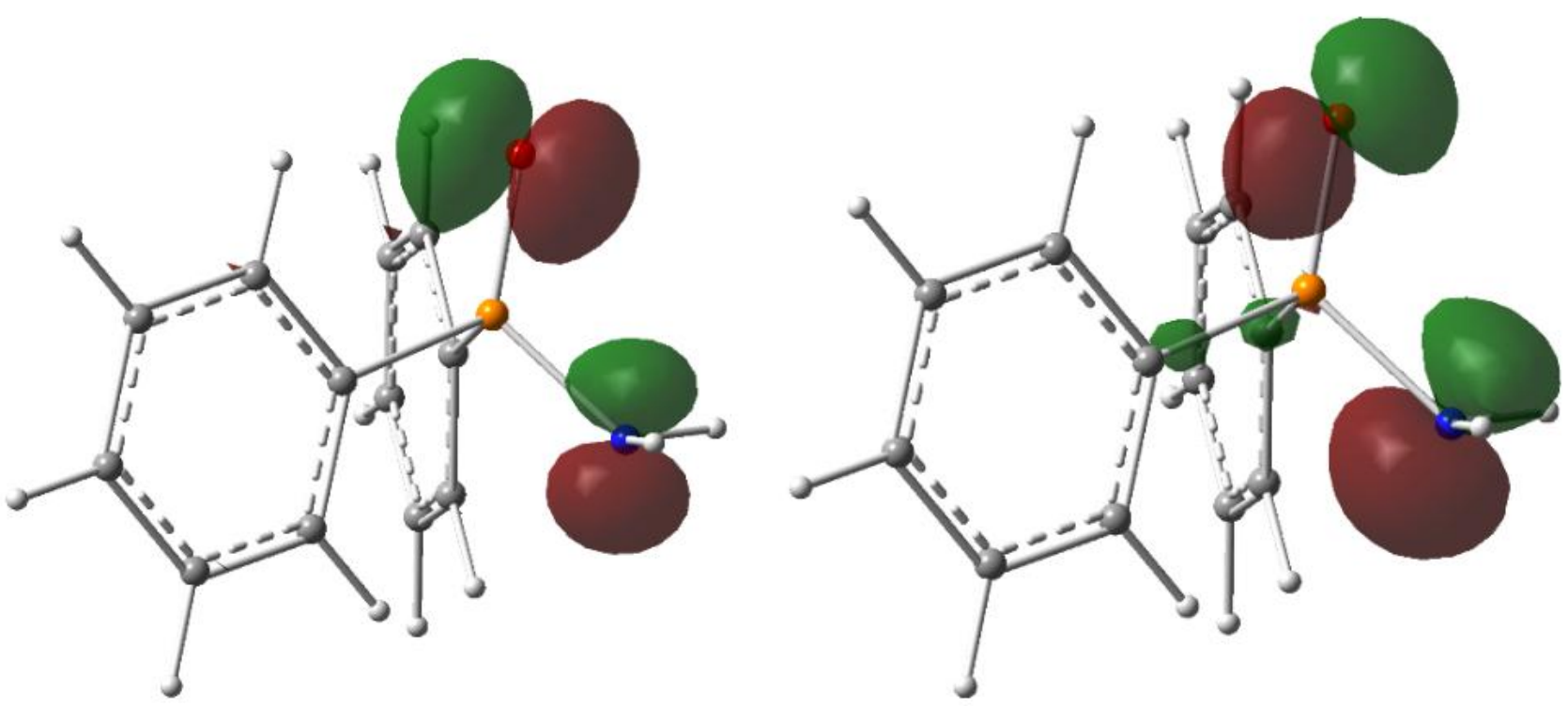

Figure S9. DFT optimized structure of O=P$(Ph)_2NH_2$ with superimposed the contour plots of π type orbitals with significant amplitudes at the N atom (HOMO – 2 and HOMO – 6). The P=O bond coincides approximately with the line of intersection of the two phenyl planes. The H-N-H'-P angle of –129° (H and H' are the hydrogen atoms of NH_2) shows that H lies outside the PNH' plane (Table S4).

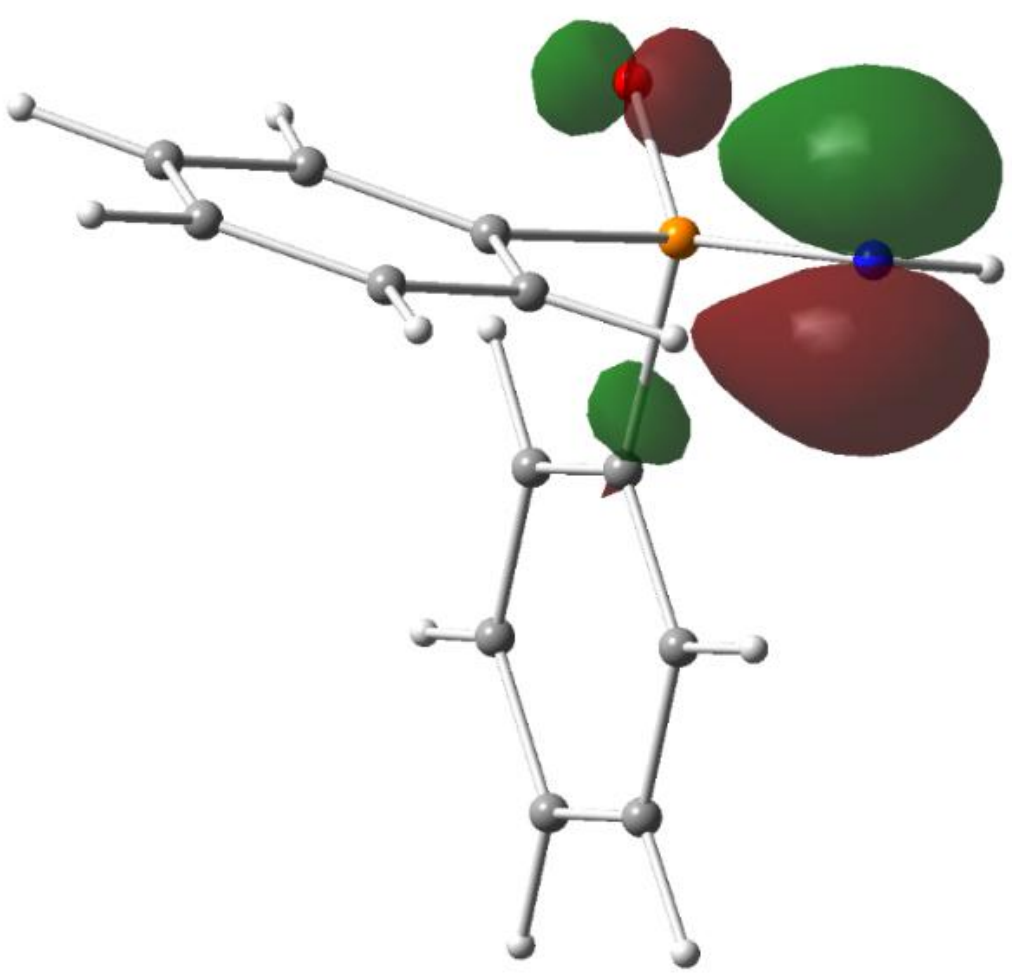

Figure S10. DFT optimized structure of $[O=P(Ph)_2NH]^-$, mimicking a deprotonated ligand arm of $[poat]^{3-}$, with superimposed the contour plots of the HOMO which has the localized features of a lone pair π orbital at the N atom.

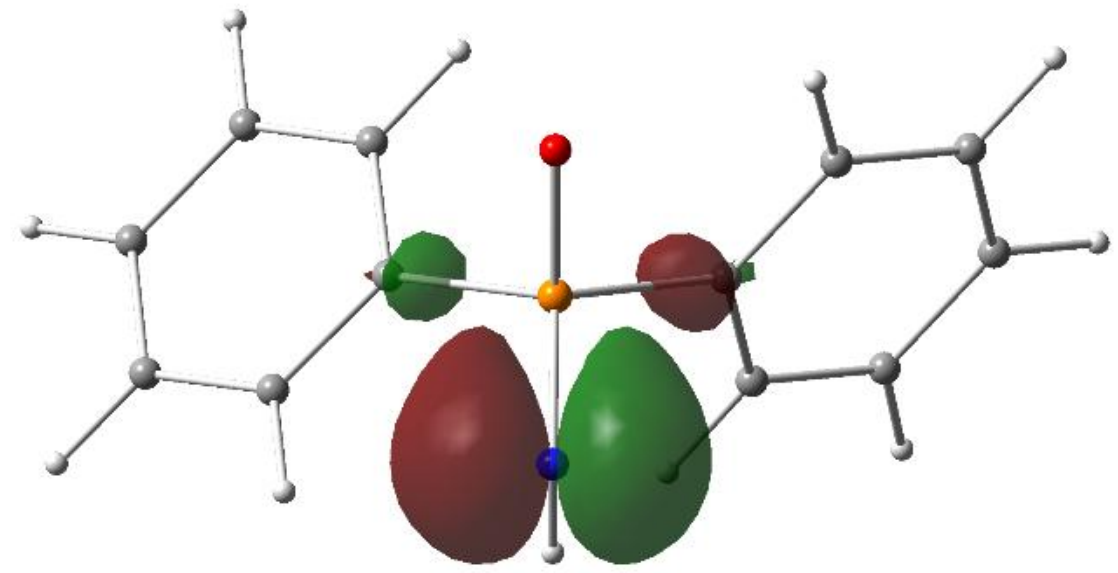

Figure S11. DFT structure of $[O{=}P(Ph)_2NH]^-$ optimized under the constraint that dihedral O-P-N-H angle is fixed to 180°. Superimposed is the contour plot of the HOMO, which has the features of a lone pair π orbital localized at the N atom, like the one in Figure S10. The structure is viewed along an axis which corresponds to an equatorial Fe-N coordination bond of $[poat]^{3-}$-bound iron in the dinuclear complex (N.B. The deleted H and remaining NH of the model replace Fe and NC in $[Fe^{m+}poat]^{n+}$, respectively).

Comments on the H-atom abstraction mechanism

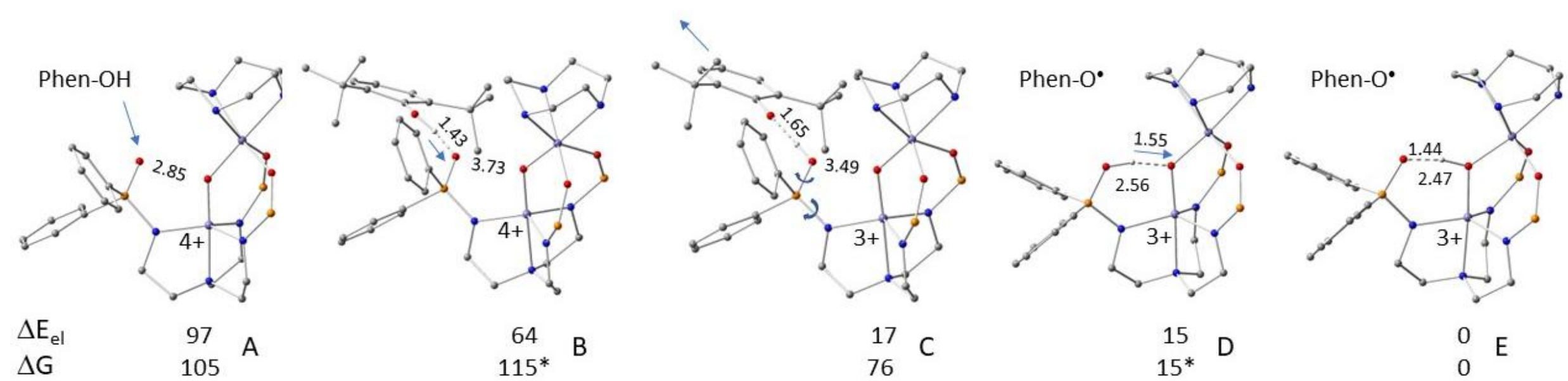

Figure S12. Intermediates of the proposed mechanism for H-atom abstraction from 2,6-di-^{t}Bu-phenol by $[(TMTACN)Fe^{IV}(O)Fe^{III}poat]^{2+}$ (**8**) (A) based on DFT calculations. From left to right, the DFT structures and electronic (ΔE_{el}, in kJ/mol) and 298 K free energies (ΔG, in kJ/mol) relative to final product $[(TMTACN)Fe^{III}(OH)Fe^{III}poat]^{2+}$ (E). All energies include the substrate or product, 2,6-di-^{t}Bu-phenoxyl radical. B–D are intermediates. Energies with an asterisk are estimates and not from frequency calculations. For clarity, only the hydrogen atom involved in the transfer is displayed and ligand phenyl and methyl groups have been removed. The $O_P \cdots O_{bridge}$ and H···O distances of H-bonds (dashed) are indicated in Å. The blue arrows indicate the motions that lead to the next structure in the sequence. Color code: H (white), C (grey), N (blue), O (red), P (yellow), Fe (grey-blue).

The first intermediate (Figure S12B) was created by constraining the phenolic proton distance to the O-atom of the phosphinic amido arm to a value of 1.43 Å during the optimization. Without this constraint, the optimization converts the first intermediate to the second intermediate (Figure S12C). The free energy of the first intermediate is an estimate because of this constraint (see next paragraph). Similarly, for the second intermediate, the distance between the proton (now on the O-atom of the phosphinic amido arm) to the oxido bridge was constrained to 1.55 Å during the optimization, again preventing its conversion to the third intermediate (Figure S12D) during optimization. The value of ΔG for the first intermediate (Figure S12B) is now slightly greater than for the initial state, which we associate with a small activation free energy barrier of 10 kJ/mol for the overall reaction.

The electronic (ΔE_{el}) and free (ΔG) energies of the initial state (Figure S12A) relative to the final state (Figure S12E), which both represent states in which the substrate is not bound to the complex, are roughly equal. However, the free energy of the second intermediate (Figure S12C) relative to the final state (76 kJ/mol) is much higher than the ΔE_{el} value (17 kJ/mol) due to a reduction in the translational entropy contribution to ΔG in the substrate-bound intermediate. This contribution has been estimated to be 51 kJ/mol from the energies given in Figure S12, using the expression $\Delta E_{el}(A) - \Delta E_{el}(C) - [\Delta G(A) - \Delta G(C)]$, and has been used to estimate the ΔG for the first intermediate (Figure S12B) by adding it to ΔE_{el} = 64 kJ/mol, yielding the value ΔG = 115 kJ/mol listed with an asterisk. As both the third intermediate (Figure S12D) and the final state (Figure S12E) are not bound to the substrate, we have estimated the relative free energy of the third intermediate (Figure S12D) using $\Delta G \approx \Delta E_{el}$. As a result of these considerations, the ΔG for the first intermediate (Figure S12B) is now slightly greater than for the initial state (Figure S12A), effectively turning the first intermediate into a transition state associated with a small activation free energy barrier of 10 kJ/mol.

Table S6. Experimental Mössbauer quadrupole splittings (mm/s) for the Fe^{II} ions of complex **4** as a function of temperature.

T (K)	ΔE_Q POAT (δ, 1.03)	ΔE_Q TMTACN (δ, 1.22)
4	3.20	2.34
50	3.19	2.34
100	3.12	2.27
150	3.05	2.19

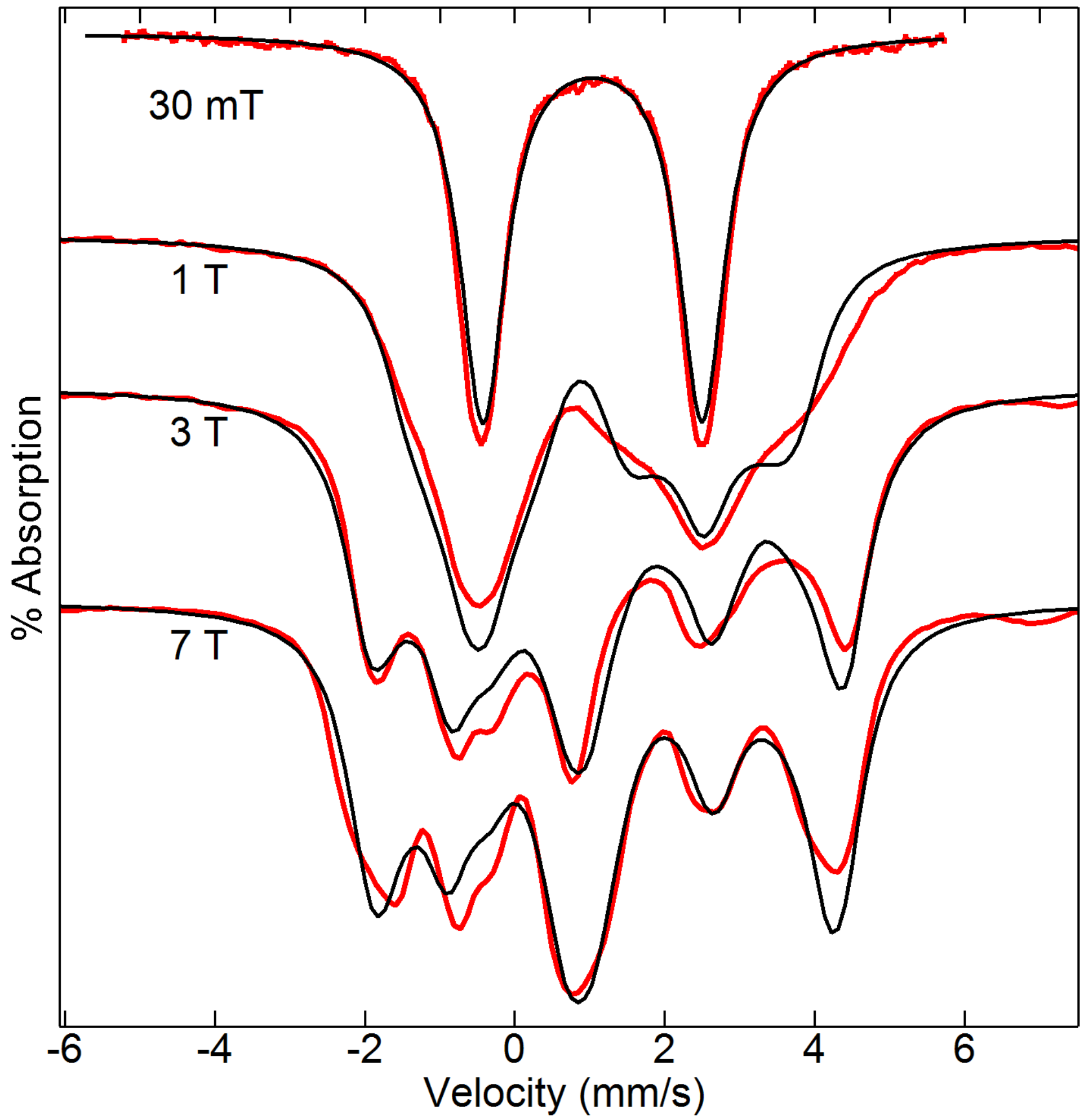

Figure S13. Mössbauer spectra (red traces, A-D) of K_2[**2**] in powder recorded at 4.2 K and the magnetic fields as listed. The black traces are global least-square fits for a S = 2 Fe^{2+} species with parameters given in Table 1.
